# Actin Structure-Dependent Stepping of Myosin 5a and 10 during Processive Movement

**DOI:** 10.1371/journal.pone.0074936

**Published:** 2013-09-19

**Authors:** Jianjun Bao, Daniel Huck, Laura K. Gunther, James R. Sellers, Takeshi Sakamoto

**Affiliations:** 1 Department of Physics and Astronomy, Wayne State University, Detroit, Michigan, United States of America; 2 Department of Physiology, School of Medicine, Wayne State University, Detroit, Michigan, United States of America; 3 National Heart, Lung, and Blood Institute, National Institutes of Health, Bethesda, Maryland, United States of America; German Cancer Research Center, Germany

## Abstract

How myosin 10, an unconventional myosin, walks processively along actin is still controversial. Here, we used single molecule fluorescence techniques, TIRF and FIONA, to study the motility and the stepping mechanism of dimerized myosin 10 heavy-meromyosin-like fragment on both single actin filaments and two-dimensional F-actin rafts cross-linked by fascin or α-actinin. As a control, we also tracked and analyzed the stepping behavior of the well characterized processive motor myosin 5a. We have shown that myosin 10 moves processively along both single actin filaments and F-actin rafts with a step size of 31 nm. Moreover, myosin 10 moves more processively on fascin-F-actin rafts than on α-actinin-F-actin rafts, whereas myosin 5a shows no such selectivity. Finally, on fascin-F-actin rafts, myosin 10 has more frequent side steps to adjacent actin filaments than myosin 5a in the F-actin rafts. Together, these results reveal further single molecule features of myosin 10 on various actin structures, which may help to understand its cellular functions.

## Introduction

Myosin 10 is an unconventional myosin with processive motility when dimerized [1-3]. It is enriched at the tips of filopodia and other cellular protrusions with dynamic actin reorganization such as lamellipodia, invadopodia and membrane ruffling [4-8]. Myosin 10 is involved in filopodial formation and undergoes intra-filopodial movement, which suggests the possibility that it functions as a component of the intra-filopodial transport system [4-6,9]. Myosin 10 is also required for angiogenesis, neuron development and proper spindle assembly via binding with microtubules [10-14].

Like other members of the myosin superfamily, myosin 10 is composed of three domains: a conserved N-terminal motor domain, which binds actin filament and ATP, a neck domain which serves as a rigid lever arm to facilitate the power stroke and a C-terminal tail containing three PH domains, one MyTH4 domain and one FERM domain which serves to target the protein inside cells [5,7]. The neck of myosin 10 contains three IQ motifs each of which can bind one calmodulin (CaM) or CaM-like proteins, followed by a single α-helical (SAH) domain [15]. The SAH domain effectively extends the length of the neck to be about 27 nm which is similar to the length of the neck of the highly processive motor myosin 5a, which contains six IQ motifs [15]. The sequence comprising this SAH domain was initially thought to form a coiled-coil [5,16], but subsequent studies showed that neither truncated fragments of myosin 10 containing this sequence or full length myosin 10 in cells readily formed dimers [8,17]. Moreover subsequent studies showed that full length myosin 10 is a single-headed motor but can be independently converted into active dimer when the monomers are brought into close proximity by clustering on the surface of intracellular cargoes or actin filaments [3,9].

Processive movement is an important feature for motor proteins to transport cargo in cells. Among the 35 classes of myosins identified thus far [18], vertebrate myosin 5a was the first myosin shown to move continuously along actin filaments as a single molecule without dissociation (i.e processive movement) [19,20]. The processivity of this double-headed molecular motor has been intensively studied *in vitro*. Single molecule experiments using electron microscopy [21], optical trapping [19,22-25], total internal reflection fluorescence (TIRF) microscopy [20,26-30], and atomic force microscopy (AFM) [31] demonstrated that myosin 5a takes 36 nm steps along actin filaments in a hand-over-hand fashion and solution transient kinetics studies confirm that the kinetic mechanism of myosin-5 is appropriate for a processive motor [32]. Fewer or more than six IQ motifs in the neck lead to step size shorter or longer than 36 nm, respectively [25,33].

Unlike the well-characterized myosin 5a, the single molecule studies on the processivity of myosin 10 remain controversial. One study showed that myosin 10 preferred to move processively along F-actin bundled with fascin rather than on single actin filaments, and took a much shorter step size of 18 nm on the bundles [1,2]. By contrast, the work from another group showed little or no selectivity of myosin 10 for F-actin bundles [3]. Moreover, the step sizes of myosin 10 on the bundles include both 34 nm and 20 nm steps, with the shorter step size being attributed to a side step onto an adjacent actin filament within a bundle [3]. These studies used myosin 10 molecules that differed in the structure of the tail region. The former group used GCN4 leucine after the SAH domain [1,2], while the latter group used the coiled-coil of myosin 5a [3]. To clarify the processive movement of myosin 10 on different actin structures, we used a fluorescently-labeled single myosin 10 heavy meromyosin (HMM) construct that was forced into dimerization by a leucine zipper as well as a myosin 5a HMM and studied their detailed stepping mechanisms on a single actin filament and two-dimensional (2-D) F-actin rafts with different F-actin bundling proteins.

We show that myosin 10 moves processively on both single actin filaments and F-actin rafts with a step size of 31 nm. On 2-D F-actin rafts, myosin 10 prefers to move more processively on fascin-bundled F-actin rafts. Moreover, both myosins exhibit side stepping to adjacent actin filament on fascin-F-actin rafts, but not on α-actinin-F-actin rafts.

## Materials and Methods

### Protein expression and preparations

Rabbit skeletal muscle actin aceton powder was purchased from Pel-Freez Biological and actin was prepared as described previously [34]. The cDNA plasmid encoding human fascin was a gift of Dr. Fumio Matsumura (Rutgers University, NJ) and fascin protein was purified from *E. coli* with GST fusion tag [35]. The GST tag was cleaved by thrombin after purification and fascin protein without GST tag was used in this study. α-actinin purified from chicken gizzard was obtained from Sigma-Aldrich. Mouse myosin 5a HMM (m5-HMM) was prepared as described previously [36]. The cDNA encoding bovine myosin 10 HMM fragment (1-940 amino acids) was amplified by PCR followed by sub-cloning into pFastBac1 vector (Invitrogen). The GCN4 leucine-zipper sequence and the Flag epitope tag (DYKDDDDK) were engineered before the stop codon. m5-HMM or myosin 10 HMM with leucine-zipper (m10-HMM-Zip) was co-expressed with calmodulin using the baculovirus Sf9 expression system [33]. The expressed proteins were purified from Sf9 cell pellets by anti-FLAG-affinity chromatography [3]. Endogenous calmodulin of m5-HMM and m10-HMM-Zip were replaced by Alexa Fluor 488-labeled bovine calmodulin at a ratio of 5: 1 (Alexa488-CaM: myosin) as described previously [37]. In some experiments, a higher ratio of 50: 1 was used.

### Single molecule motility assay

Single molecule motility assays were performed with TIRF microscopy on an IX81 microscope (Olympus America). Briefly, two diode pumped laser lines (473 nm and 556 nm, OEM Laser, UT) for TIRF illumination were introduced from a side port of a cube turret in the IX81. Both lasers were individually focused at a back-focal plane in the TIRF objective lens (Apo N, x60, N.A. 1.49 oil, Olympus America). Image magnifying lenses (2x and 4x) were used in front of an electron-multiplier CCD camera (EMCCD, Hamamatsu). Rhodamine phalloidin- and 10% biotin-labeled F-actin was bound onto the cover-glass via a biotin/avidin system. m5-HMM or m10-HMM-Zip with Alexa Fluor 488-CaM solution in the motility buffer containing 20 mM MOPS (pH 7.4), 5 mM MgCl_2_, 0.1 mM EGTA, 40 mM KCl, 1 µM CaM, and 50 mM dithiothretol was then added with various concentrations of ATP as indicated. An oxygen scavenging system containing 25 µg/ml glucose oxidase, 45 µg/ml catalase, and 2.5 mg/ml glucose was also used as described previously (7). All experiments were performed at 23 °C controlled by a home-made enclosed box with a heat controller (Air-Therm, World Precision Instrument, Inc., FL). The motility of myosin molecules was captured and analyzed with Metamorph (Molecular Devices) and IDL softwares (ITT visual information).

### Preparation of two dimensional (2-D) F-actin rafts

The preparation of 2-D F-actin rafts cross-linked by fascin or α-actinin was based on a previously described method [38-41]. Briefly, actin polymerization buffer (50 mM KCl, 20 mM PO_4_, 1mM ATP, 2mM MgCl_2_, 0.02% β-mercaptoethanol, 1 mM EGTA, 0.05% NaN_3_) was first added to a Teflon well. A phospholipid solution (2 µl) composed of a 3: 7 w/w mixture of dilauryl phosphatidylcholine (DLPC) and didodecylmethyl ammonium (DDMA) in chloroform was pipetted into the well to form a lipid monolayer (Langmuir-Blodgett film) on the surface. This mixture was incubated for 60 minutes at 4 °C. Globular actin (30 µg/ml) mixed with the actin-bundling protein fascin (11 µg/ml) or α-actinin (40 µg/ml) was then added carefully underneath of the surface of the lipid monolayer and incubated for 6 hours at 4 °C or 4 hours at 23 °C. The 2-D F-actin paracrystalline bundle arrays that were formed underneath the lipid monolayer were transferred to a coverslip and labeled with rhodamine-phalloidin or phalloidin for 10 minutes at 4 °C. The coverslip was carefully attached to a slide-glass using double-sided tape, forming a flow-cell for single motility assay. To quantify the number of actin filament in the rafts, the intensity of the rhodamine-labeled F-actin rafts in the region of interest was normalized to the intensity of rhodamine-phalloidin-labeled single actin filament with the same size of the region of interest. The average number of actin filaments in fascin- and α-actinin-F-actin rafts were 3.98 ± 1.8 (n = 102, mean ± S.D.) and 4.88 ± 1.85 (n = 89, mean ± S.D.), respectively (Figure S1).

### Electron Microscopy

For electron microscope imaging, 2-D F-actin paracrystalline arrays underneath Langmuir-Blodgett film were transferred to 400-mesh carbon-coated copper grids (Electron Microscopy Sciences), stained with 2% aqueous uranyl acetate for 30 sec, and the grid was washed twice with motility buffer as described above. Transmission electron microscopy (TEM) images were taken at 100 keV with 20,000 and 35,000 magnifications (TEM, JEOL-2010, JEOL). The center-to-center distance between actin filaments in the 2-D rafts was measured as described [38,41]. Briefly, images were processed using a fast Fourier transform (FFT) filter to detect spatial periodicity of actin filament (MetaMorph, Molecular Devices). Line scan intensity was measured across the filtered image of the rafts and then fit to a sum of Gaussian distributions using the maximal likelihood method [37]. The distance between the peaks of these distributions represents the distance between the centers of adjacent actin filaments.

### Tracking and analysis of myosin movement

Particle localization was achieved at near-nanometer resolution using FIONA [26]. The tracking of fluorescence spots were performed using custom IDL routine works as described [42]. All tracks less than two seconds in duration were eliminated to remove non-processive as diffusive movements. A custom Origin C routine was used to calculate the total run length, which is the length between the starting point and the end point of the movement, and the total displacement, which is the sum of the individual displacements (frame by frame) during a track. The average velocity of the individual displacements was calculated from the data. Tracks with run lengths less than 250 nm were also filtered out. These criteria were strong indicators of a non-processive myosin movement. After software filtering, each track that met these criteria was manually analyzed to determine if it was in fact a processive myosin movement. To calculate the percent of attachment that were moving, a separate analysis using the IDL routine was carried out to calculate the number of processive runs and the total number of attaching/dissociating myosin molecule.

## Results

### Myosin 10 exhibits less processive motility than myosin 5a along single actin filaments

A GCN4 leucine zipper, which is a dimeric coiled-coil [43] was added to the myosin 10 sequence following amino acid 940 to create a dimeric HMM-like fragment that was used in this study. To visualize the myosin molecule, we replaced endogenous calmodulin with Alexa Fluor 488-labeled calmodulin (Alexa488-CaM) [33]. Photo-bleaching experiments showed that the average numbers of Alexa488-dye was 1.9 per calmodulin and the average number of Alexa488-dye was 3.9 ± 2.4 per 10-HMM-zipper (m10-HMM-Zip), and 4.4 ± 2.9 per myosin 5-HMM (m5-HMM), meaning that about two Alexa488-CaM molecules were exchanged into one myosin molecule (Figure S2). In addition, the distribution of the number of fluorophore on myosins showed single Gaussian (Figure S2 D), and no extremely bright fluorescent spot was detected on m5-HMM and m10-HMM-Zip under TIRF microscopy, indicating that most of the myosin 5 and 10 molecules used in our study were dimers, but not oligomers or aggregated.

**Figure 1:**
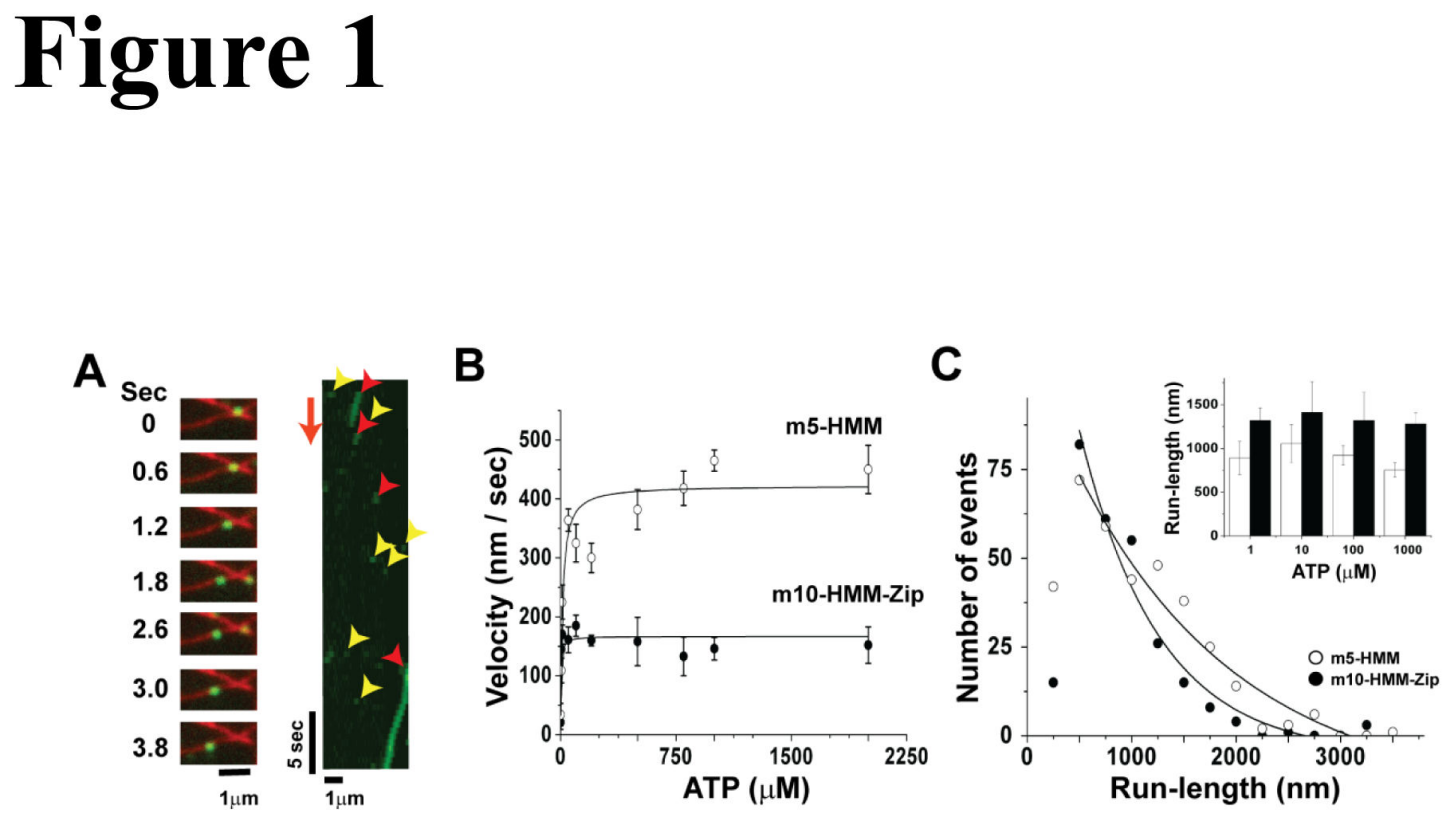
Single molecule motility assays of myosins 10 and 5a along single actin filaments. (A) Still images from a movie showing movement of single molecules of m10-HMM-zip along a single actin filament. A series of images on the left panel shows the actin filament (red) and the fluorescently-labeled m10-HMM-zip in green at the indicated times. The images on the right are kymographs of the data depicted on the left. Red arrow heads show the moving m10-HMM-zip molecules and the yellow arrow heads show the molecules which attached and detached from actin without significant movement. (B) Velocity of single m5-HMM (open circle) and m10-HMM-Zip (closed circle) molecules at various ATP concentrations. Maximum velocities of m5-HMM and m10-HMM-Zip are 421 ± 38 nm/sec and 218 ± 29 nm/sec, respectively (mean ± S.D.). The data were fit to the Michael-Menten equation. (C) The run length distribution of m5-HMM (open circle) and m10-HMM-Zip (closed circle) at 1 mM ATP. Run length data were fit with single exponential equation (solid line). The mean run lengths of m5-HMM and m10-HMM-Zip were 1221 ± 255 nm and 755 ± 85 nm, respectively (mean ± S.D.). Data points from runs < 250 nm were omitted from the fits. The inset is the run-length of m5-HMM (closed bar) and m10-HMM-Zip (open bar) against ATP concentrations. Error bar is standard deviation (S.D.).

Both m10-HMM-Zip and m5-HMM moved processively along single actin filaments as observed by TIRF microscopy. Sequential images and kymographs of m10-HMM-Zip are shown in Figure 1A and Movie S1. The maximum velocity of m10-HMM-Zip was much less than that of m5-HMM (190 ± 40 nm/sec vs. 420 ± 38 nm/sec, mean ± S.D., Figure 1B). Moreover, the mean run-length of m10-HMM-Zip (755 ± 88 nm, n=264, mean ± S.D., Figure 1C, closed circles) was shorter than that of m5-HMM (1221 ± 255 nm, n=349, mean ± S.D., Figure 1C). In addition, at 1 mM ATP (saturating), 89% of m5-HMM and 35% of m10-HMM-Zip attachments to actin resulted in measurable processive movements, indicating that most of attached m5-HMM move processively along single actin filament while about 65% of m10-HMM-Zip bound and dissociated from single actin filament without significant movement (Table 1). Examples of such transient, nonproductive interactions are shown in Figure 1A yellow arrowheads.

**Table 1:** The run-length (top) and the percentage (bottom) of the moement of m10-HMM-Zip and m5-HMM on single actin filaments and F-actin rafts.

		*2D-F-actin rafts*	
*myosin*	*Single F-actin*	*Fascin*	*α-actinin*
m10-HMM-Zip	755 ± 88 nm 35.0% (158/452)	695 ± 89 nm 58.8% (3074/5233)	1465 ± 125 nm 23.5% (1963/8355)
m5-HMM	1221 ± 255 nm 89% (289/325)	1255 ± 135 nm 65.8% (4148/6382)	998 ± 87 nm 72.3% (3536/7835)

Run-length (nm): mean ± standard deviation. The number of moving spots per total observation spots which bound and dissociated during the observation window is shown in parenthesis (no. of moved spots / no. of total observation spots).

We also examined the effects of ATP concentration (1 µM to 1mM) on the processive movement (Figure 1C inset). The mean run-lengths of both m10-HMM-zip and m5-HMM were not significantly changed with ATP concentrations, though the mean run-length of m10-HMM-zip at 10 µM ATP was slightly longer than at other ATP concentrations.

### Myosin 10 walks hand-over-hand manner along actin filaments with a step size of 30.6 nm

**Figure 2:**
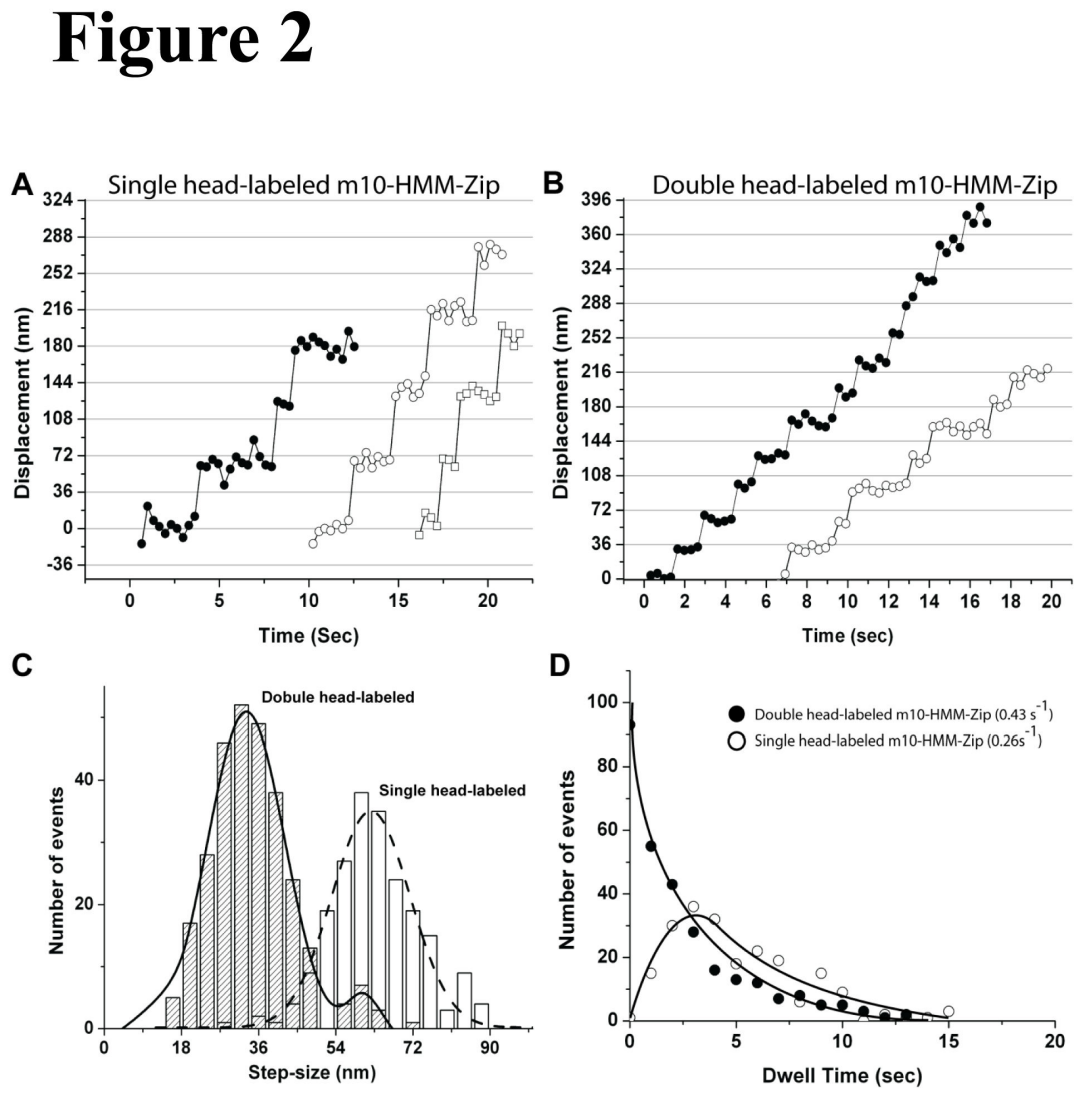
Analysis of processive movement of m10-HMM-Zip on single actin filament. (A and B) Representative stepping traces of single head-labeled (A) and double-head-labeled (B) m10-HMM-Zip. Each data point represents a 0.5 s acquisition time window. (C) Histogram of the step sizes of m10-HMM-Zip. Bars show distribution of the step size for single head-labeled m10-HMM-Zip (open bar) and double-head labeled m10-HMM-Zip (shaded bar). The solid line was fit with the double-head labeled m10-HMM-Zip to a sum of two Gaussian distribution using maximal likelihood [37]. The dashed line shows the fit to the data with a single Gaussian. The multi-modal fit was justified as described previously [37]. The value for this fit between two peaks was *p* < 0.03. The peaks of the step size distribution for double-head labeled m10-HMM-Zip (mean ± SD) are 31.4 ± 10.2 nm and 61.5 ± 6.5 nm (n = 287). The peak of the step size distribution for single head-labeled m10-HMM-Zip (mean ± SD) is 60.8 ± 8.8 nm (n = 210). (D) Dwell time distribution of m10-HMM-Zip. The dwell time of double-head labeled m10-HMM-Zip (closed circle) was fit to a single-exponential equation, P(t) = k exp(-kt). Dwell time of single head-labeled m10-HMM-Zip (open circle) was fit to an equation on a hand-over-hand model equation; P(t) = tk^2^exp(-kt) [26]. The rate constants of the fits are 0.43 s^-1^ (single head-labeled m10-HMM-Zip) and 0.26 s^-1^ (double-head labeled m10-HMM-Zip).

The step size of myosin 5a and myosin 10 along single actin filament was analyzed by FIONA [26] at 0.3 µM ATP for m5-HMM and 1 µM ATP for m10-HMM-Zip. As the ATPase rate [17,44] and velocity of myosin 10 were slower than those of myosin 5a (Figure 1A, Figure S2A), a lower concentration of ATP (0.3 µM) resulted in sufficiently slow movement of myosin 5a so that sufficient number of photons could be collected for identification of individual steps, while a higher concentration of ATP (1 µM) allowed fluorescently-labeled myosins 10 to take sufficient steps before photobleaching for analysis of step-size and dwell time. Both myosins exhibited two classes of step sizes, long ones and ones where the stepping distance were approximately one half that of the long ones (Figure 2 and Figure S3). Consistent with our previous studies [45], we assigned the long stepping traces to be from molecules in which only one of the two heads of the HMM was labeled with Alexa Fluor 488. In these traces the distance that the labeled rear head traveled as it dissociated and moved to find a new forward binding site was being measured. This would be followed by a “silent” step where the unlabeled trail head detaches and reattaches in the forward position while the labeled (originally) forward head remains attached at a fixed position. The short stepping traces represent molecules where both heads were labeled with Alexa Fluor 488 and therefore the movement of the center-of-mass is measured each time either of the heads takes a step. The stepping traces of single- and double-head-labeled m10-HMM-Zip and m5-HMM are shown in Figure 2 A and B, and Figure S3 A and B. Consistent with previously published studies [26,37,45], our experiment also showed that myosin 5a moves in a hand-over-hand manner with a step size of 36 nm (Figure S3). However, myosin 10 took shorter steps at low ATP concentration (Figure 2). When the step size of single- and double-head-labeled m10-HMM-zip (Figure 2 C) were fit with a single Gaussian and the sum of two Gaussians, respectively, the double-head-labeled m10-HMM-Zip was shown to take a step size (the center of mass) of 30.6 ± 11.2 nm (n = 287, mean ± S.D.). In cases where only a single head of m10-HMM-Zip was labeled, the step size was 63.3 ± 10.7 nm (n = 210, mean ± S.D.), approximately twice as large for double head-labeled m10-HMM (Figure 2 C). Additionally, about 6% of the double head-labeled m10-HMM-zip took a step of 61 nm, which probably represents either the sum of two fast steps where an obvious intermediate dwell was not found or where the dye on one of the two heads photo-bleached at the end (Figure 2 C right-shaded-bars). When the exchange ratio was increased to 50: 1 of Alexa488-CaM to myosin, the step-size histograms of m5-HMM and m10-HMM-Zip showed predominantly short step-sizes (36-nm and 30-nm, respectively, Figure S4 A and B), indicating that most molecules were double-head-labeled.

A plot of the dwell-time distributions supports this interpretation of the short and long steps. The distribution of dwell times for the long stepping events was best fit by the sum of two exponentials as expected for the case where every other step would be “silent” [26,37]. The detachment rate constant determined from the fit (0.26 s^-1^, Figure 2 D open circles) is approximately half that of the double-head-labeled m10-HMM-Zip (0.43 s^-1^, Figure 2 D close circles), confirming only half of the steps were observed with single-head labeled HMM since movements of the unlabeled head would be silent. The above results suggest that the two heads of myosin 10 move alternatively along single actin filaments, supporting the hand-over-hand model of myosin 10 movement [3]. Of note, the distribution of the step size of m10-HMM-Zip (S.D. = 16 nm) (Figure 2 C) was slightly wider than that of m5-HMM (S.D.: 9 nm, Figure S3 C), which may result from the flexibility of the SAH domain located between the third calmodulin-binding site and the GCN4 leucine zipper.

### Myosin 10 moves more processively on fascin-F-actin rafts than on α-actinin-F-actin rafts

**Figure 3:**
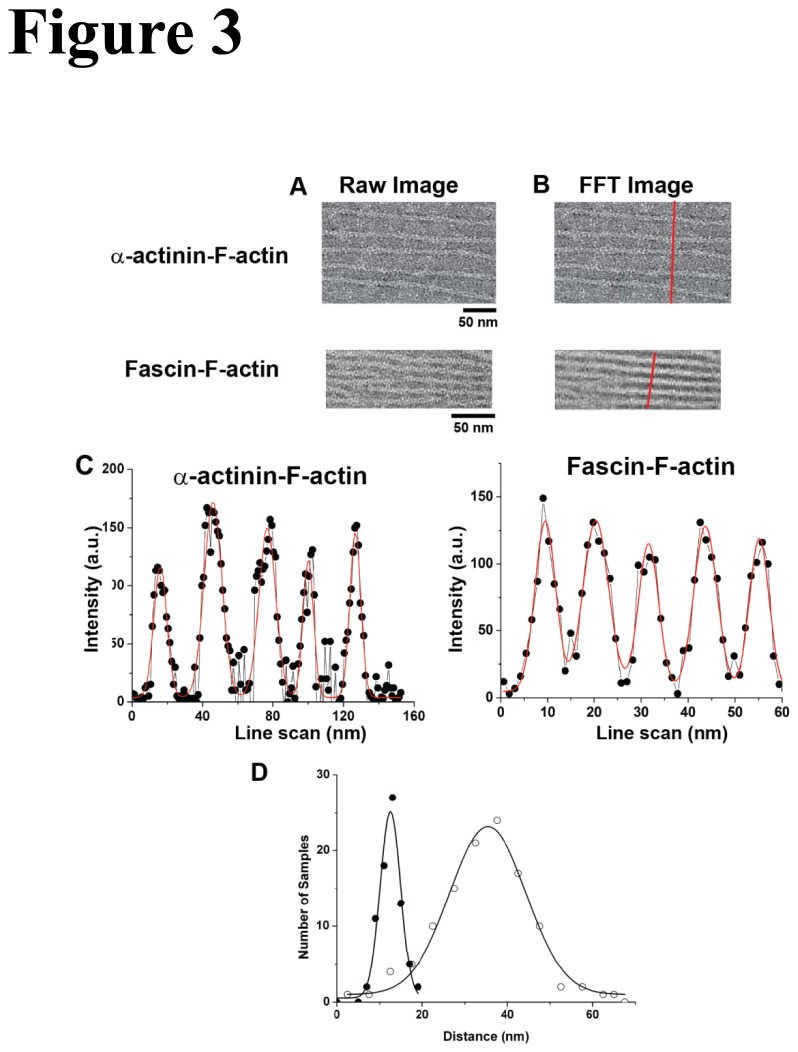
Analysis of the electron micrograph of fascin- and α-actinin-F-actin rafts. (A) Unprocessed electron microscope images of actin rafts created with α-actinin (top) or fascin (bottom). (B) Same images transformed with a fast Fourier transform (FFT) filter, a line scan (red line) analysis of the intensities. (C) Intensity profile from the line scan in (B). The data were fit with sum of Gaussians (red lines). The peaks represent the position of actin filaments in the rafts. (D) Histogram of the center-to-center distance between actin filaments with fascin (closed circle) and α-actinin (open circle) analyzed with single Gaussian distribution (solid line). The average distances of fascin- and α-actinin-F-actin rafts were 12.5 ± 2.3 nm (n = 78, mean ± S.D.) and 39.7 ± 16.5 (n = 97, mean ± S.D.), respectively.

We next examined the processive motility of m10-HMM-zip and m5-HMM on 2-D F-actin rafts on a lipid monolayer that were cross-linked by either α-actinin or fascin [38,40,41], both of which were reported to form parallel F-actin bundles on the 2-D F-actin rafts [40,46]. The alignment of actin filaments in 2-D rafts were visualized by electron microscopy (Figure 3) and the center-to-center distance between actin filaments was analyzed using a fast Fourier transform (FFT) filter to detect spatial periodicity. In an α-actinin-F-actin raft, the inter-filament distance is 35 nm while this is 12 nm in a fascin-F-actin raft (Figure 3 D), which is consistent with previous studies [38,41,47,48].

**Figure 4:**
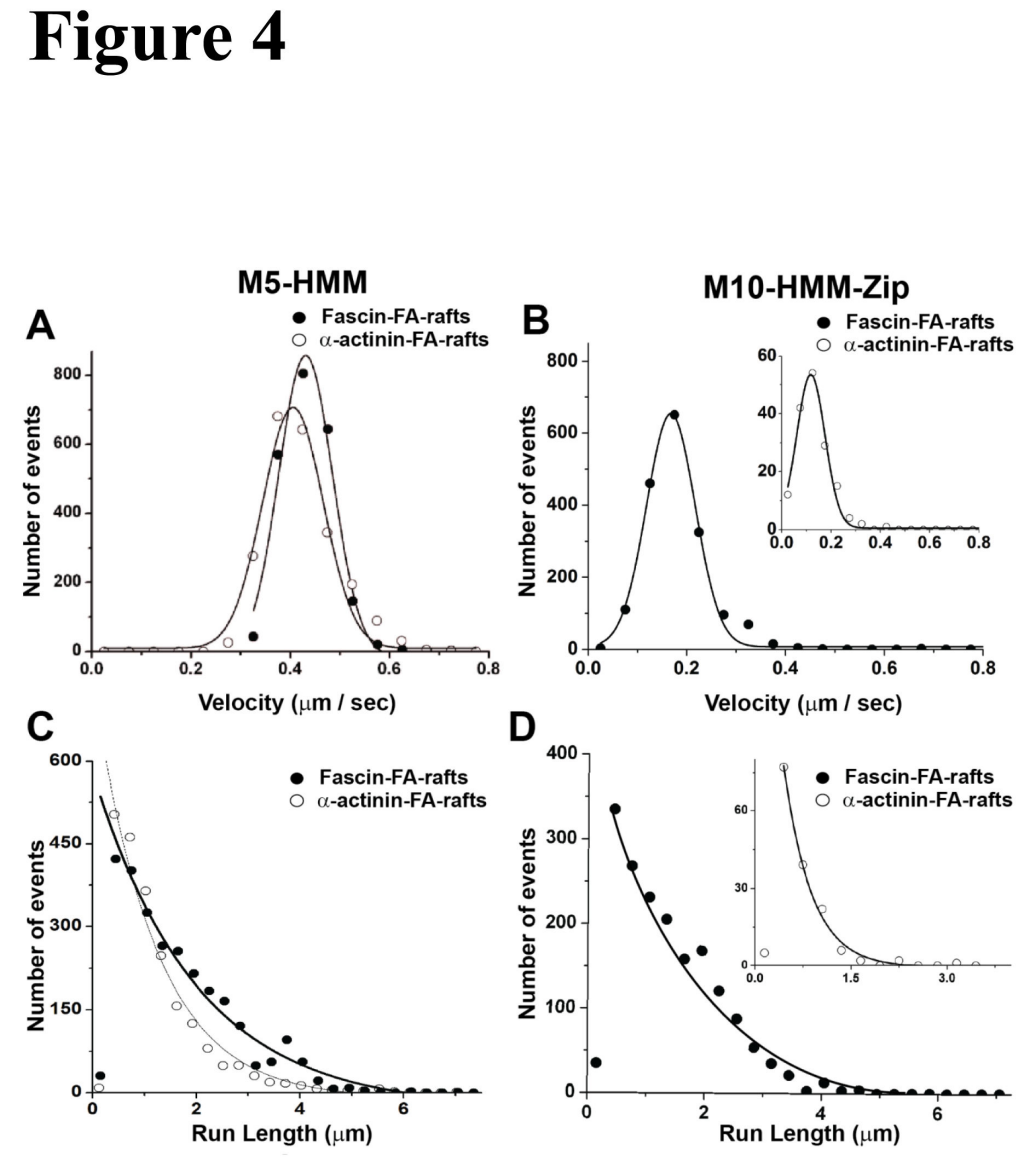
Velocity of m5-HMM and m10-HMM-Zip on 2-D actin rafts with fascin and α-actinin. (A and B) The velocity distributions of m5-HMM (A) and m10-HMM-Zip (B) on 2D F-actin (FA) rafts with α-actinin (open circle) and fascin (closed circle). Solid lines represent a single Gaussian fit to the data. The average velocities of m5-HMM on α-actinin- and fascin-F-actin rafts were 398 ± 88 nm/sec (n = 2355) and 430 ± 144 nm/sec (n = 2200), respectively. The average velocities of m10-HMM-Zip on α-actinin- and fascin-F-actin rafts were 124 ± 36 nm/sec (n = 157) and 165 ± 43 nm/sec (n = 1633), respectively. (C and D) The run-length of m5-HMM (C) and m10-HMM-Zip (D) on 2D-actin rafts with α-actinin (open circle) and fascin (closed circle). The run-lengths of m5-HMM on F-actin rafts with α-actinin (open circle) and fascin (closed circle) were 998 ± 87 nm (n = 2335, means ± S.D.) and 1255 ± 135 nm (n = 2200, means ± S.D.), respectively. The run-lengths of m10-HMM-Zip on F-actin rafts with α-actinin and fascin were 564 ± 23 nm (inset: open circle, n = 157, means ± S.D.) and 1465 ± 125 nm (closed circle, n = 1633, means ± S.D.), respectively.

Single molecule motility assay showed different degrees of processivity of m10-HMM-Zip and m5-HMM moving on α-actinin- and fascin-F-actin rafts, demonstrating that the type of track could influence the movement (Figure 4, Figure S5, Movies S2, S3, S4, and S5). This could be manifest in both the tendency to move processively and in the run length. First, when the same concentration of m5-HMM and m10-HMM-Zip was used, only 23.5% of m10-HMM-Zip molecules that attached to the α-actinin-F-actin rafts moved processively while 58.8% of the attached molecules moved on fascin-F-actin rafts (Table 1). In contrast, m5-HMM showed no such selective movement on α-actinin-F-actin rafts and fascin-F-actin rafts (65.8% vs. 72.3%, Table 1). The average velocity of m10-HMM-Zip with 1 mM ATP on α-actinin-F-actin rafts (124 ± 36 nm/s, mean ± S.D, n = 157, Figure 4 B inset) and on fascin-F-actin rafts (165 ± 43 nm/s, mean ± S.D., n = 1633, Figure 4 B) were similar to that obtained using single actin filaments as the track. Under the same condition, the average velocities of m5-HMM with 1mM ATP on α-actinin-F-actin rafts and fascin-F-actin rafts were 398 ± 88 nm/s (mean ± S.D, n=2335, Figure 4 A open circles) and 430 ± 144 nm/s (mean ± S.D, n = 2200, Figure 4 A close circles). Both were similar to that the velocity on single actin filaments (420 ± 38 nm/s, Figure 1 B). Second, the run-length of m10-HMM-Zip on α-actinin-F-actin rafts was significantly shorter than on fascin-F-actin rafts (695 ± 89 nm vs. 1465 ± 125 nm, mean ± S.D.), whereas the run-lengths of m5-HMM on fascin- and α-actinin-F-actin rafts were similar, 1255 ± 135 nm and 998 ± 87 nm, respectively (Figure 4 C and D, Table 1). These results suggest that fascin-F-actin rafts favor the processivity of m10-HMM-Zip.

### Distinct stepping patterns of myosin 10 on fascin- and α-actinin-F-actin rafts

**Figure 5:**
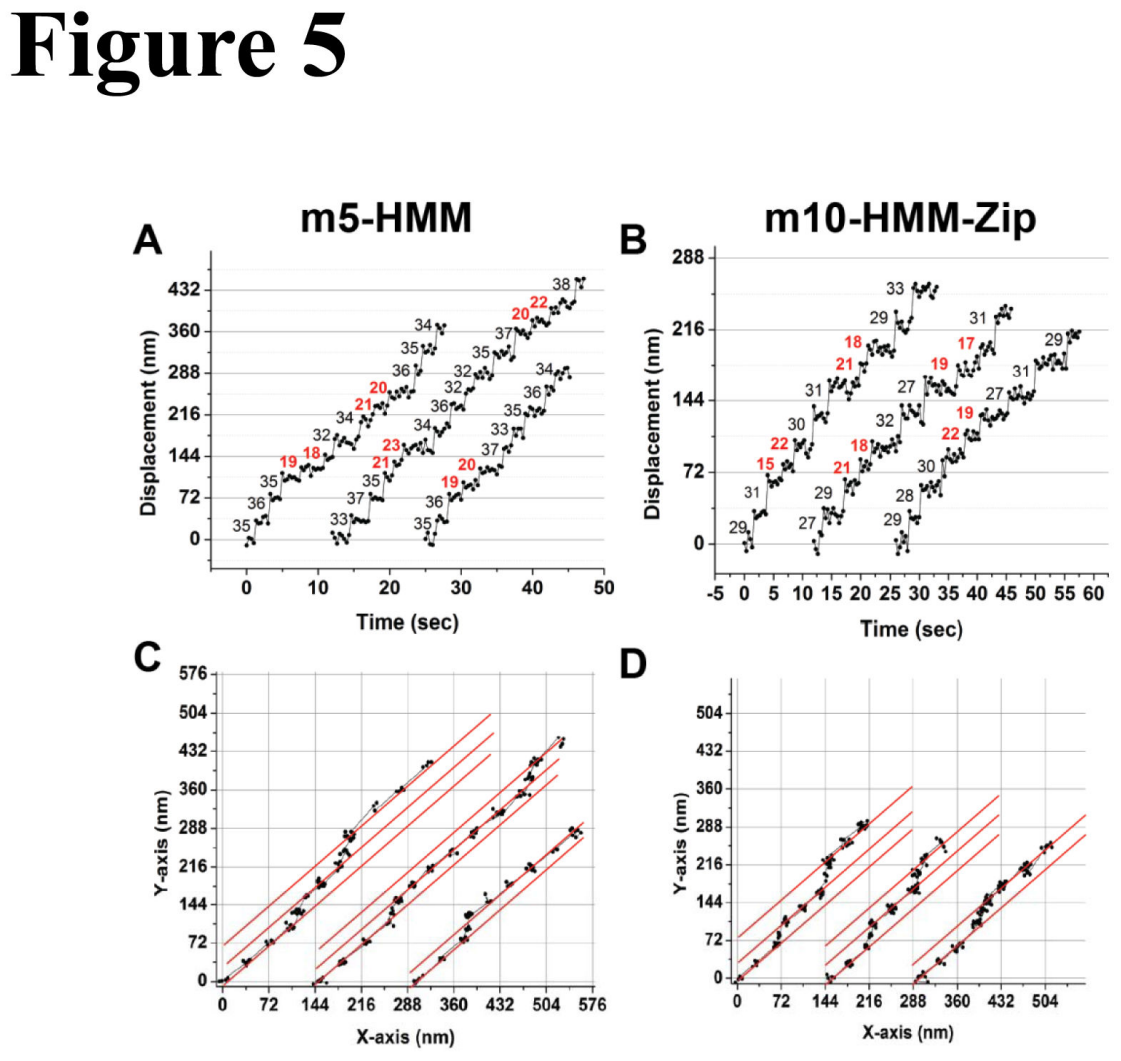
Stepping traces of double-head labeled m5-HMM and double-head labeled m10-HMM-Zip on F-actin rafts with fascin. Stepping traces of m5-HMM (A) and m10-HMM-Zip (B) over time. (C) XY-plots of the stepping positions of m5-HMM data shown above in panel (A). (D) XY plots of the stepping positions of m10-HMM-Zip data shown above in panel (B). Small step sizes (red numbers in A and B) represent the side-steps from one actin filament to the other on the F-actin rafts. Red solid lines in C and D indicate 45 degree angles with respect to the X-axis along each actin filament in the rafts.

**Figure 6:**
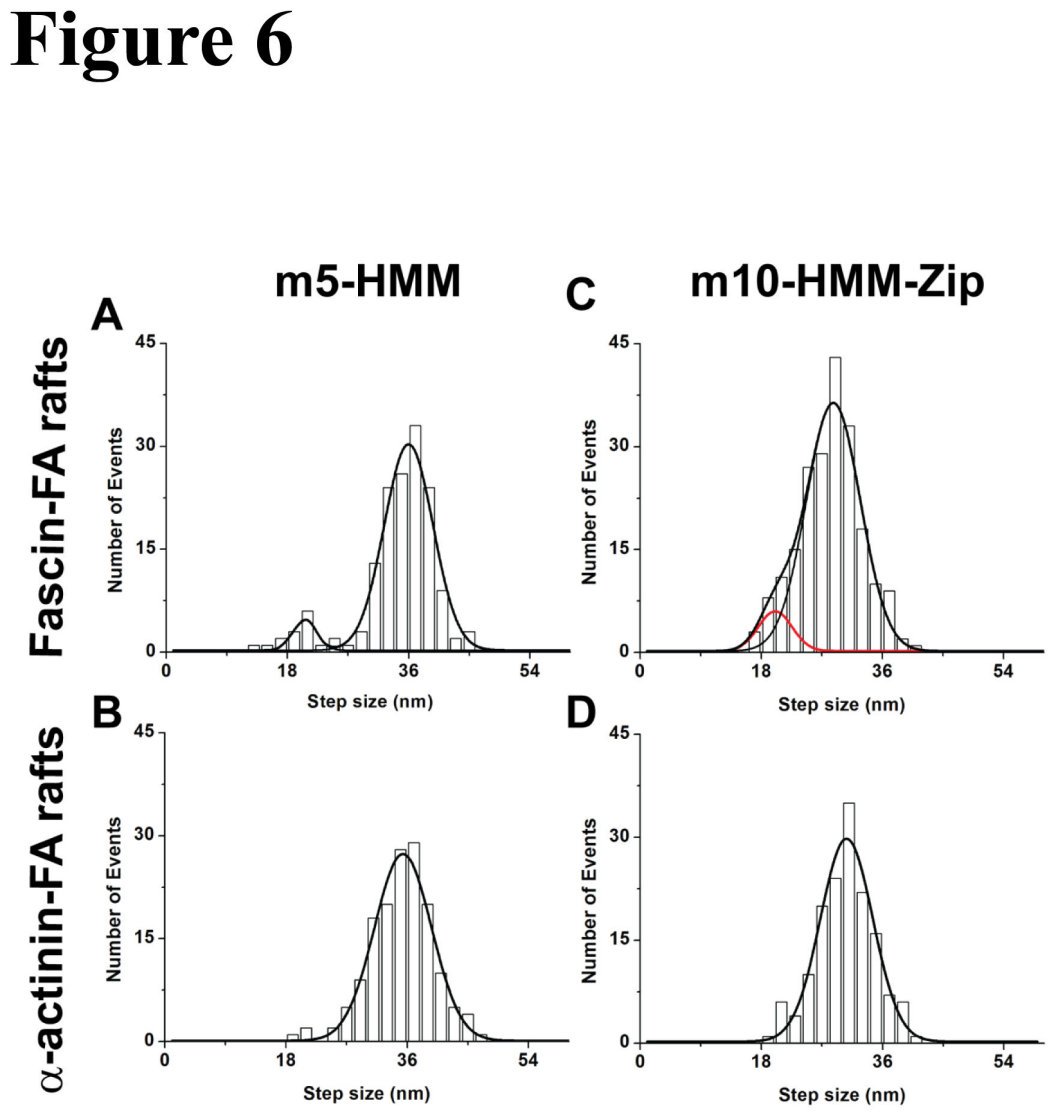
Distribution of step sizes for double-head labeled m5-HMM and double-head labeled m10-HMM-Zip on 2D-F-actin rafts bundled with fascin and α-actinin. Distribution of step sizes for the movement of m5-HMM on fascin-F-actin rafts (A) and on α-actinin rafts (B). Distribution of step sizes for the movement of m10-HMM-Zip on fascin-F-actin rafts (C) and on α-actinin rafts (D). The solid lines in panels A and C represent the fit of the data with a sum of two Gaussian distribution using maximal likelihood as described above. Red line in (C) represents the fit to the shorter step size. The solid lines in panels B and D represent the fit with single Gaussian curve. The peaks of step size of m5-HMM were (A) 36.2 ± 6.3 nm and 21.6 ± 3.5 nm (n = 158, means ± S.D.) on fascin-F-actin rafts and (B) 35.1 ± 8.3 nm (n = 154, means ± S.D.) on α-actinin-F-actin rafts. The peaks of step size of m10-HMM-Zip were (C) 30.8 ± 6.8 nm and 21.3 ± 3.8 nm (n = 209, means ± S.D.) and (D) 31.5 ± 4.8 nm (n = 153, means ± S.D.).

**Figure 7:**
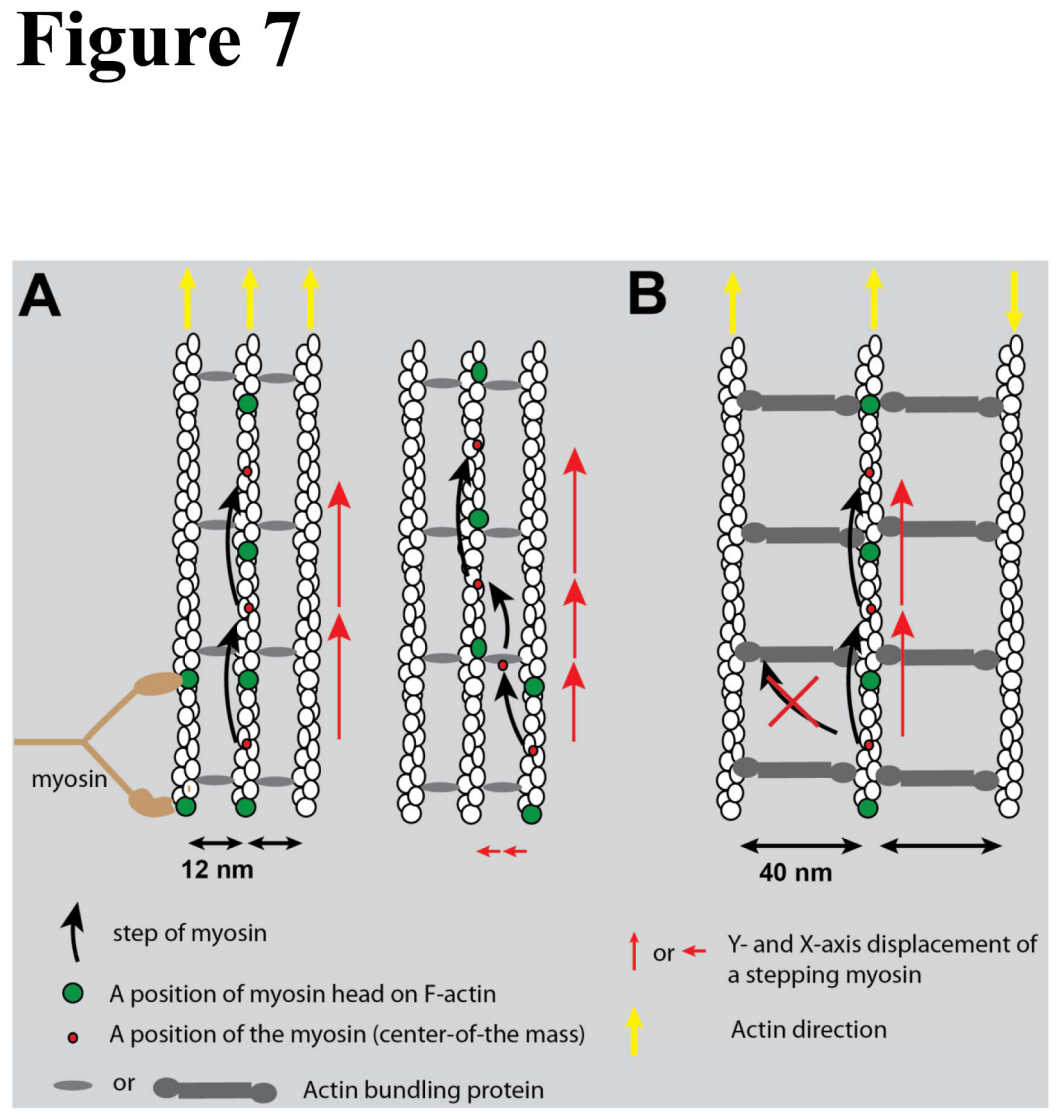
Stepping model of myosin on actin rafts. Myosin stepping model on F-actin rafts. Two heads of myosin (light brown color) bind on an actin monomer (green) in actin filaments. The center of the mass of the myosin is depicted by a red circle. When myosin steps forward (black arrow), the detached head binds a new actin monomer (green). (A) The model for stepping on fascin-F-actin rafts. Left-hand side shows the myosin molecule stepping forward along an actin filament in the rafts. The length of the red arrow represents the magnitude of the step-size. Black arrows represent the direction of the stepping. Right-hand side model shows a myosin molecule taking a side step to an adjacent actin filament in the rafts. Note that when a myosin takes a side step onto an adjacent actin filament the center of mass movement takes two short steps. Yellow arrow represents the direction of actin filament in the rafts. (B) The model of stepping on α-actinin-F-actin rafts. A myosin molecule cannot reach an adjacent actin filament and thus cannot have a side step.

The stepping behavior and step size of m10-HHM-zip and m5-HMM on the above 2D-F-actin rafts were analyzed by FIONA. When a myosin 5 molecule moved along fascin-F-actin rafts, most of its steps were 36.2 nm, while 14% of its steps were shorter with a value of 21.6 nm (Figures 5 A and 6 A). The shorter step could be correlated in space to a side step to an adjacent parallel actin filament. Each side step should be marked by two back-to-back shorter steps since the center of mass of these double-head labeled molecules are being followed (Figure 7). This means that m5-HMM takes one side step out of every seven total steps. On α-actinin-F-actin rafts, the step size of myosin 5 exhibited a single peak at 35.1 nm, consistent with the wider spacings of the actin filaments (Figure 6 B). By contrast, the major step size of m10-HMM-Zip on fascin-F-actin rafts was 30.8 nm, while 28% of the step size was 21.3 nm (Figures 5 B and 6 C). Similar to myosin 5-HMM, m10-HMM-Zip showed a major step size of 31.5 nm without detectable side steps on α-actinin-F-actin rafts (Figure 6 D). These results suggest that m10-HMM-Zip took more frequent side-steps on fascin-F-actin rafts than did m5-HMM. We also determined the direction of the myosin step on fascin-F-actin rafts according to the fluorescence intensity in both vertical and lateral directions (XY coordinates, Figure 5 C and D), confirming that most m5-HMM and m10-HMM-Zip moved straight on actin filaments (red-straight line), but occasionally took shorter side steps with right-hand direction against the direction of the F-actin rafts.

## Discussion

In cells actin is present in a pleomorphic variety of structures including from single actin filaments, branched filament networks, loose meshworks, and bundles. There are many F-actin bundling proteins that create bundles with different interfilament spacing and the actin filaments might be either parallel or antiparallel when formed *in vitro* depending on the bundling protein and the conditions. Our goal in this study was to determine how myosin 5a and 10 move on different actin structures. We showed that myosin 10 moved processively along both single actin filaments and F-actin rafts with 31 nm step sizes. We further demonstrated that myosin 10 moved more processively on fascin-F-actin rafts than on single filaments or on α-actinin-F-actin rafts, whereas myosin 5a showed comparable processive movement along both single filaments and F-actin rafts. Finally, myosin 5a and 10 were found to take side steps occasionally while walking along the actin filament in fascin-F-actin rafts.

The proteins used in this study were across many species. The amino acid sequences of myosin 5a and 10 are well conserved among the mammalian species including the ones used in our studies, thus we believe that our results still reflect the common properties of myosin 5a and 10 beyond the specific species. Other proteins including actin, fascin, α-actinin, and calmodulin are also well conserved among the species used in this study. Regardless of their potential specific functions and regulations in specific species, they share the general functions in building the common actin structures that utilized in this study. In addition, the species-specific proteins that we have used allow us to directly compare our results (run lengths, velocity, etc) to the large volume of results performed in our lab and others. Similarly, due to the availability of abundant protein resources, skeletal muscle alpha-actin instead of beta- or gamma-actin was used in this study as well as most of the studies in the same field. Actins are highly conserved among the isoforms both in their amino acid sequences as well as their biological functions with respect to their polymerization to filaments. Note that De La Cruz et al [49] examined the actin isoform dependence of myosin 5a kinetics and found little difference. This study is, in general, looking at the broad question of how the myosins move on actin bundles and is less concerned with small differences in rates that might come about from the use of different actin isoforms.

Single molecule motility assays were performed to assess the processivity of the two-headed m10-HMM-Zip along F-actin filaments. We did not test the processivity of myosin 10 with single head on actin filaments, as there was no evidence that a single-headed myosin 10, which did not possess a secondary actin binding site, would be able to move processively. Compared to the highly processive motor myosin 5a [23,24,26,28,33], our results showed that myosin 10 was less processive. Only 35% of m10-HMM-Zip that initially attached to actin in the current study was shown to move processively along single actin filament for a measurable distance, while nearly 90% of attached myosin 5 molecules exhibited processive motility. Moreover, the run length of myosin 10 molecules was shorter than myosin 5, and the maximum velocity was slower than myosin 5a. The velocity of m10-HMM-Zip obtained in this study is in agreement with the data from other laboratories [1-3]. However, the run length of myosin 10 in our study was about 750 nm [1,3]. Nagy et al reported a much shorter run length of 170 nm [1], whereas Sun et al obtained a longer run length of 950 nm [3]. The difference in run length measurement may originate from the species of myosin 10 used in the studies and the structure of the forced myosin 10 HMM dimer, such as the length of coiled-coil region, the joint structure between neck region and the coiled-coil region and solution conditions, which are pH, ionic strength, and temperature. We used a GCN4 leucine-zipper at C-terminal fused to the myosin 10 construct after amino acid 940, while other groups used either the same GCN4 leucine-zipper at C-terminal but fused after amino acid 920 [1,2] or fused a portion of the coiled-coil tail of myosin 5a after 1042th amino-acid of myosin 10 [3]. These factors may affect the run-length and step size. The step size (31 nm) of m10-HMM-Zip in our study was shorter than that of m5-HMM (36 nm), and this is in agreement with the maximum velocity and actin activated ATPase rate of myosin 10, that is, 30.6 (nm) x 5.5 (/s) = 168 (nm/sec). Moreover, the shorter step size of myosin 10 may explain its shorter run length.

Second, we characterized the processive movement of myosin 5 and myosin 10 on highly ordered F-actin structures by using the 2-D F-actin rafts with α-actinin and fascin to control the center-to-center distance between the actin filaments in the rafts. We found that the run length and the number of movements of m10-HMM-Zip on the fascin-F-actin rafts was more than that on α-actinin-F-actin rafts, suggesting that myosin 10 prefers to move on fascin-F-actin rafts, which is consistent with the observation of a previous study [1] and the subcellular localization of myosin 10 at the tips of filopodia, where F-actin is mainly cross-linked by fascin [50,51]. By contrast, myosin 5a shows no preference for either single filaments, or F-actin rafts cross-linked by fascin or α-actinin [1,2].

A substantial portion of the myosin 10 did not start walking on actin filaments or actin rafts when observe under TIRF microscopy at saturating ATP concentrations. There are several possible reasons for the higher number of abortive binding events with myosin 10 than with myosin 5. First, it is possible that a myosin must bind with a proper stereochemical position on actin in order to commence a processive run and it is possible that myosin 5 is more successful in doing this than myosin 10. Second, since these measurements are not done under FIONA conditions, a very short processive run of only two or three steps would likely not be detected as a processive run. Given that myosin 10 is inherently less processive than myosin 5 (as shown by the shorter run length) such short runs might be more frequently “observed” with it and scored as a nonprocessive event.

On fascin-F-actin rafts, myosin 10 took mostly 31 nm steps along with a more occasional 21 nm side step, and myosin 5a took mostly 36 nm steps with less frequent 22 nm side steps. This stepping pattern is consistent with a previous report, except that the step size of myosin 10 on fascin-F-actin bundle (27 nm) was shorter than that on single actin filament (34 nm) [3]. However, our results contrast with that of Nagy et al who reported a uniform 18 nm step size on fascin-F-actin bundle [1]. Our results support a stepping model where both myosins take multiple processive steps along an actin filament within the bundle and occasionally take a side step to an adjacent actin filament (Figure 7). In this model, the two heads of myosin bind on single actin filament in the F-actin rafts and occasionally straddle two adjacent actin filaments when taking a side step. This differs from the model proposed by Nagy et al where myosin-10 was suggest to move along an actin bundle with its two heads “waddling” between two adjacent actin filaments.

We did not observed side-steps of m5-HMM and m10-HMM-Zip on α-actinin-F-actin rafts, suggesting that the heads of both myosin molecules could not reach adjacent actin filaments in these rafts due to the wider distances (40 nm) between the center-to-center of actin filaments. However, a recent study showed that myosin 5a could take a side step or switch to another actin filament on a three-dimensional α-actinin actin bundle [52]. In this case, the actin filaments within the bundle were of mixed polarity and this resulted in bi-directional movements of individual myosin 5a molecules within the bundle [52].

There are many actin binding proteins that might create road blocks or obstacles to movement along a single actin filament. Two mechanisms might allow for myosins to dodge these obstacles in order to continue moving processively. Electron microscopic images of myosin 5a bound to actin in the presence of ATP shows that myosin 5a prefers to bind with its two motor domains 13 actin monomers (~36 nm) apart when moving along a single actin filament which effectively allows it to walk “straight” and avoid spirally around the filament [21,53]. However, the lead motor can also bind to the 11^th^ or 15^th^ actin monomer which might allow the molecule to take a shorter or longer step if the preferred actin monomer is blocked. There is no comparable electron microscopic information on the interaction of myosin 10 with actin in the presence of ATP. The ability of myosins 5a and 10 to take side steps on fascin-actin rafts provides a second method to avoid obstacles that might prevent the unbound myosin head from attaching to its preferred actin monomer when searching for a new actin binding site. Of note, 2-D actin rafts was used to facilitate the detailed analysis of the stepping manners of myosin 5 and 10 along individual actin filaments by eliminating the complicated structures in 3-D bundles, as seen in cells. As such, the 2-D rafts may not provide the exact road blocks similar to those mentioned above that referred to cellular conditions. However, it provides a simplified, alternative approach to study the 3-D bundles that are difficult structures to study the stepping manner along single filaments.

## Supporting Information

Figure S1 **Fluorescence intensity measurement of fascin- and α-actinin-F-actin rafts.** (A) Actin filaments were labeled with rhodamine-phalloidin. The total fluorescence in the rectangle region of interest (white rectangle) was measured and from the background fluorescence was subtracted to get the fluorescence intensity of a single actin filament. The intensities of fascin- and α-actinin-F-actin rafts were normalized to that of a single actin filament. (B) Histogram of the normalized intensity of fascin- (closed circle) and α-actinin- (open circle)-F-actin rafts. The solid (fascin-F-actin rafts) and dashed (α-actinin-F-actin rafts) lines represent the fit with single Gaussian curve. The average number of actin filaments in fascin- and α-actinin-F-actin rafts were 3.98 ± 1.8 (n = 102, mean ± S.D.) and 4.88 ± 1.85 (n = 89, mean ± S.D.), respectively.
(TIF)

Figure S2 **Determination of the number of Alexa Fluor 488 dyes per HMM molecule.** Individual molecules of Alexa488-CaM-m10-HMM-Zip (A), Alexa488-CaM-m5-HMM (B), and Alexa488-labeled calmodulin (C) were bound on a cover glass surface and imaged by TIRF microscope. The photo-bleaching events were observed as a function of time. Step-wise photo-bleaching events were observed (red lines). (D) Histogram of the number of photo-bleaching events per molecule of m10-HMM-Zip, m5-HMM, and CaM. Data were fit with a single Gaussian curve. The average number of Alexa Fluor 488 on m10-HMM-Zip, m5-HMM, and CaM are 3.9 ± 2.4 and 4.4 ± 2.9, and 1.9, respectively.
(TIF)

Figure S3 **Single molecule analysis of m5-HMM stepping along single actin filaments.** (A and B) Stepping traces of single- and double-head labeled m5-HMM, respectively. (C) Step size histogram of m5-HMM. The data of single-head-labeled m5-HMM (open bar) were fit with single Gaussian curve. The average step size is 72 ± 9 nm (n = 175, mean ± S.D.) The data of double-head labeled m5-HMM (right-hand shade) were fit with the sum of two Gaussians. The major and minor peaks are 36.5 ± 12 nm and 72.8 ± 4.7 nm (n = 270, mean ± S.D.), respectively. (D) The dwell time distribution of single- (open circle) and double- (closed circle) head labeled m5-HMM. The dwell time of single-head-labeled m5-HMM (0.21 s^-1^) was half of double-head labeled m5-HMM (0.43 s^-1^). The method for the fitting curves was described in Figure 2 D.
(TIF)

Figure S4 **Step-size histograms of m5-HMM and m10-HMM-Zip with high exchang ratio of Alexa488-CaM to myosin.** The step-size of myosin 5 and myosin 10 at low ATP concentration was measured under high exchange ratio of Alexa488-CaM to myosin molecule (30:1). (A) The step-size histogram of the double- (n = 308, right-hand shade) and single-head-labeled (n = 28, open bar) m5-HMM. The data of single- and double-head-labeled m5-HMM were fit with single Gaussian (solid line) and the sum of two Gaussians (dashed line), respectively. The average step-size of single-head-labeled m5-HMM is 72 ± 6.7 nm. The major and minor peaks of step-sizes of double-head-labeled m5-HMM step-size are 36.8 ± 5.2 nm and 73.3 ± 7.0 (mean ± S.D.). (B) The histogram of the step-size of double- (n = 344, right-hand shade) and single- head-labeled m10-HMM-Zip (n = 33, open bar). The data of single- and double-head-labeled m10-HMM-Zip were fit with single Gaussian curve (solid line) and the sum of two Gaussians (dashed line), respectively. The average step-size for double-head-labeled m10-HMM-Zip is 60.8 ± 3.3 nm. The major and minor step-size of single-headed-labeled m10-HMM-Zip are 30.2 ± 4.2 nm and 61.0 ± 5.6 nm, respectively.
(TIF)

Figure S5 **Movement of m5-HMM and m10 HMM-Zip on fascin-F-actin and α-actinin-F-actin rafts.** In all panels the left image shows 2D bundle of F-actin labeled with rhodamine phalloidin and colored red. The yellow lines in each of these images were analyzed for the kymographs which are shown in the right panels. (A and B) Fluorescence images of fascin-F-actin rafts and kymographs showing movement of m5-HMM (A) and m10-HMM-Zip (B) (C and D) Fluorescence images of α-actinin-F-actin rafts and kymographs showing movements of m5-HMM (C) and m10-HMM-Zip (D). In the kymograph, red arrowheads represent processive movement and yellow arrowheads represent non-processive movement, i.e. where the myosin molecule bound and dissociated without significant movement.
(TIF)

Movie S1 **The movements of myosin 10-HMM-Zip (m10-HMM-Zip) along single actin filaments.** All movies were captured by two frames/second and are 2.5 times faster than real speed.
(AVI)

Movie S2 **The movements of m5-HMM on fascin-F-actin rafts.**
(AVI)

Movie S3 **The movements of m5-HMM on α-actinin-F-actin rafts.**
(AVI)

Movie S4 **The movements of m10-HMM-Zip on fascin-F-actin rafts.**
(AVI)

Movie S5 **The movements of m10-HMM-Zip on α-actinin-F-actin rafts.**
(AVI)

## References

[1] Nagy S, Ricca BL, Norstrom MF, Courson DS, Brawley CM et al. (2008) A myosin motor that selects bundled actin for motility. Proc Natl Acad Sci U S A 105: 9616-9620. doi:10.1073/pnas.0802592105. PubMed: 18599451. 18599451 10.1073/pnas.0802592105 PMC2474510

[2] Ricca BL, Rock RS (2010) The stepping pattern of myosin X is adapted for processive motility on bundled actin. Biophys J 99: 1818-1826. doi:10.1016/j.bpj.2010.06.066. PubMed: 20858426. 20858426 10.1016/j.bpj.2010.06.066 PMC2941030

[3] Sun Y, Sato O, Ruhnow F, Arsenault ME, Ikebe M et al. (2010) Single-molecule stepping and structural dynamics of myosin X. Nat Struct Mol Biol 17: 485-491. doi:10.1038/nsmb.1785. PubMed: 20364131. 20364131 10.1038/nsmb.1785 PMC2876314

[4] Berg JS, Cheney RE (2002) Myosin-X is an unconventional myosin that undergoes intrafilopodial motility. Nat Cell Biol 4: 246-250. doi:10.1038/ncb762. PubMed: 11854753. 11854753 10.1038/ncb762

[5] Berg JS, Derfler BH, Pennisi CM, Corey DP, Cheney RE (2000) Myosin-X, a novel myosin with pleckstrin homology domains, associates with regions of dynamic actin. J Cell Sci 113 19: 3439-3451. PubMed: 10984435. 10984435 10.1242/jcs.113.19.3439

[6] Kerber ML, Jacobs DT, Campagnola L, Dunn BD, Yin T et al. (2009) A novel form of motility in filopodia revealed by imaging myosin-X at the single-molecule level. Curr Biol 19: 967-973. doi:10.1016/j.cub.2009.03.067. PubMed: 19398338. 19398338 10.1016/j.cub.2009.03.067 PMC2817954

[7] Lu Q, Yu J, Yan J, Wei Z, Zhang M (2011) Structural basis of the myosin X PH1(N)-PH2-PH1(C) tandem as a specific and acute cellular PI(3,4,5)P(3) sensor. Mol Biol Cell 22: 4268-4278. 21965296 10.1091/mbc.E11-04-0354 PMC3216653

[8] Tokuo H, Ikebe M (2004) Myosin X transports Mena/VASP to the tip of filopodia. Biochem Biophys Res Commun 319: 214-220. doi:10.1016/j.bbrc.2004.04.167. PubMed: 15158464. 15158464 10.1016/j.bbrc.2004.04.167

[9] Plantard L, Arjonen A, Lock JG, Nurani G, Ivaska J et al. (2010) PtdIns(3,4,5)P(3) is a regulator of myosin-X localization and filopodia formation. J Cell Sci 123: 3525-3534. doi:10.1242/jcs.069609. PubMed: 20930142. 20930142 10.1242/jcs.069609

[10] Zhu XJ, Wang CZ, Dai PG, Xie Y, Song NN et al. (2007) Myosin X regulates netrin receptors and functions in axonal path-finding. Nat Cell Biol 9: 184-192. doi:10.1038/ncb1535. PubMed: 17237772. 17237772 10.1038/ncb1535

[11] Yu H, Wang N, Ju X, Yang Y, Sun D et al. (2012) PtdIns (3,4,5) P3 recruitment of Myo10 is essential for axon development. PLOS ONE 7: e36988. doi:10.1371/journal.pone.0036988. PubMed: 22590642. 22590642 10.1371/journal.pone.0036988 PMC3349655

[12] Woolner S, O’Brien LL, Wiese C, Bement WM (2008) Myosin-10 and actin filaments are essential for mitotic spindle function. J Cell Biol 182: 77-88. doi:10.1083/jcb.200804062. PubMed: 18606852. 18606852 10.1083/jcb.200804062 PMC2447898

[13] Toyoshima F, Nishida E (2007) Integrin-mediated adhesion orients the spindle parallel to the substratum in an EB1- and myosin X-dependent manner. EMBO J 26: 1487-1498. doi:10.1038/sj.emboj.7601599. PubMed: 17318179. 17318179 10.1038/sj.emboj.7601599 PMC1829369

[14] Weber KL, Sokac AM, Berg JS, Cheney RE, Bement WM (2004) A microtubule-binding myosin required for nuclear anchoring and spindle assembly. Nature 431: 325-329. doi:10.1038/nature02834. PubMed: 15372037. 15372037 10.1038/nature02834

[15] Knight PJ, Thirumurugan K, Xu Y, Wang F, Kalverda AP et al. (2005) The predicted coiled-coil domain of myosin 10 forms a novel elongated domain that lengthens the head. J Biol Chem 280: 34702-34708. doi:10.1074/jbc.M504887200. PubMed: 16030012. 16030012 10.1074/jbc.M504887200

[16] Zhang H, Berg JS, Li Z, Wang Y, Lång P et al. (2004) Myosin-X provides a motor-based link between integrins and the cytoskeleton. Nat Cell Biol 6: 523-531. doi:10.1038/ncb1136. PubMed: 15156152. 15156152 10.1038/ncb1136

[17] Watanabe TM, Tokuo H, Gonda K, Higuchi H, Ikebe M (2010) Myosin-X induces filopodia by multiple elongation mechanism. J Biol Chem 285: 19605-19614. doi:10.1074/jbc.M109.093864. PubMed: 20392702. 20392702 10.1074/jbc.M109.093864 PMC2885239

[18] Odronitz F, Kollmar M (2007) Drawing the tree of eukaryotic life based on the analysis of 2,269 manually annotated myosins from 328 species. Genome Biol 8: R196. doi:10.1186/gb-2007-8-9-r196. PubMed: 17877792. 17877792 10.1186/gb-2007-8-9-r196 PMC2375034

[19] Rief M, Rock RS, Mehta AD, Mooseker MS, Cheney RE et al. (2000) Myosin-V stepping kinetics: a molecular model for processivity. Proc Natl Acad Sci U S A 97: 9482-9486. doi:10.1073/pnas.97.17.9482. PubMed: 10944217. 10944217 10.1073/pnas.97.17.9482 PMC16890

[20] Sakamoto T, Amitani I, Yokota E, Ando T (2000) Direct observation of processive movement by individual myosin V molecules. Biochem Biophys Res Commun 272: 586-590. doi:10.1006/bbrc.2000.2819. PubMed: 10833456. 10833456 10.1006/bbrc.2000.2819

[21] Walker ML, Burgess SA, Sellers JR, Wang F, Hammer JA 3rd et al. (2000) Two-headed binding of a processive myosin to F-actin. Nature 405: 804-807. doi:10.1038/35015592. PubMed: 10866203. 10866203 10.1038/35015592

[22] Veigel C, Schmitz S, Wang F, Sellers JR (2005) Load-dependent kinetics of myosin-V can explain its high processivity. Nat Cell Biol 7: 861-869. doi:10.1038/ncb1287. PubMed: 16100513. 16100513 10.1038/ncb1287

[23] Veigel C, Wang F, Bartoo ML, Sellers JR, Molloy JE (2002) The gated gait of the processive molecular motor, myosin V. Nat Cell Biol 4: 59-65. doi:10.1038/ncb732. PubMed: 11740494. 11740494 10.1038/ncb732

[24] Mehta AD, Rock RS, Rief M, Spudich JA, Mooseker MS et al. (1999) Myosin-V is a processive actin-based motor. Nature 400: 590-593. doi:10.1038/23072. PubMed: 10448864. 10448864 10.1038/23072

[25] Purcell TJ, Morris C, Spudich JA, Sweeney HL (2002) Role of the lever arm in the processive stepping of myosin V. Proc Natl Acad Sci U S A 99: 14159-14164. doi:10.1073/pnas.182539599. PubMed: 12386339. 12386339 10.1073/pnas.182539599 PMC137854

[26] Yildiz A, Forkey JN, McKinney SA, Ha T, Goldman YE et al. (2003) Myosin V walks hand-over-hand: single fluorophore imaging with 1.5-nm localization. Science 300: 2061-2065. doi:10.1126/science.1084398. PubMed: 12791999. 12791999 10.1126/science.1084398

[27] Ali MY, Krementsova EB, Kennedy GG, Mahaffy R, Pollard TD et al. (2007) Myosin Va maneuvers through actin intersections and diffuses along microtubules. Proc Natl Acad Sci U S A 104: 4332-4336. doi:10.1073/pnas.0611471104. PubMed: 17360524. 17360524 10.1073/pnas.0611471104 PMC1838602

[28] Baker JE, Krementsova EB, Kennedy GG, Armstrong A, Trybus KM et al. (2004) Myosin V processivity: multiple kinetic pathways for head-to-head coordination. Proc Natl Acad Sci U S A 101: 5542-5546. doi:10.1073/pnas.0307247101. PubMed: 15056760. 15056760 10.1073/pnas.0307247101 PMC397419

[29] Forkey JN, Quinlan ME, Goldman YE (2000) Protein structural dynamics by single-molecule fluorescence polarization. Prog Biophys Mol Biol 74: 1-35. doi:10.1016/S0079-6107(00)00015-8. PubMed: 11106805. 11106805 10.1016/s0079-6107(00)00015-8

[30] Forkey JN, Quinlan ME, Shaw MA, Corrie JE, Goldman YE (2003) Three-dimensional structural dynamics of myosin V by single-molecule fluorescence polarization. Nature 422: 399-404. doi:10.1038/nature01529. PubMed: 12660775. 12660775 10.1038/nature01529

[31] Kodera N, Yamamoto D, Ishikawa R, Ando T (2010) Video imaging of walking myosin V by high-speed atomic force microscopy. Nature 468: 72-76. doi:10.1038/nature09450. PubMed: 20935627. 20935627 10.1038/nature09450

[32] De La Cruz EM, Wells AL, Rosenfeld SS, Ostap EM, Sweeney HL (1999) The kinetic mechanism of myosin V. Proc Natl Acad Sci U S A 96: 13726-13731. doi:10.1073/pnas.96.24.13726. PubMed: 10570140. 10570140 10.1073/pnas.96.24.13726 PMC24132

[33] Sakamoto T, Wang F, Schmitz S, Xu Y, Xu Q et al. (2003) Neck length and processivity of myosin V. J Biol Chem 278: 29201-29207. doi:10.1074/jbc.M303662200. PubMed: 12740393. 12740393 10.1074/jbc.M303662200

[34] Spudich JA, Watt S (1971) The regulation of rabbit skeletal muscle contraction. I. Biochemical studies of the interaction of the tropomyosin-troponin complex with actin and the proteolytic fragments of myosin. J Biol Chem 246: 4866-4871.. 4254541

[35] Ono S, Yamakita Y, Yamashiro S, Matsudaira PT, Gnarra JR et al. (1997) Identification of an actin binding region and a protein kinase C phosphorylation site on human fascin. J Biol Chem 272: 2527-2533. doi:10.1074/jbc.272.4.2527. PubMed: 8999969. 8999969 10.1074/jbc.272.4.2527

[36] Sakamoto T, Webb MR, Forgacs E, White HD, Sellers JR (2008) Direct observation of the mechanochemical coupling in myosin Va during processive movement. Nature 455: 128-132. doi:10.1038/nature07188. PubMed: 18668042. 18668042 10.1038/nature07188 PMC2775414

[37] Sakamoto T, Yildez A, Selvin PR, Sellers JR (2005) Step-size is determined by neck length in myosin V. Biochemistry 44: 16203-16210. doi:10.1021/bi0512086. PubMed: 16331980. 16331980 10.1021/bi0512086

[38] Kitajiri S, Sakamoto T, Belyantseva IA, Goodyear RJ, Stepanyan R et al. (2010) Actin-bundling protein TRIOBP forms resilient rootlets of hair cell stereocilia essential for hearing. Cell 141: 786-798. doi:10.1016/j.cell.2010.03.049. PubMed: 20510926. 20510926 10.1016/j.cell.2010.03.049 PMC2879707

[39] Taylor KA, Taylor DW (1992) Formation of 2-D paracrystals of F-actin on phospholipid layers mixed with quaternary ammonium surfactants. J Struct Biol 108: 140-147. doi:10.1016/1047-8477(92)90013-Z. PubMed: 1486004. 1486004 10.1016/1047-8477(92)90013-z

[40] Taylor KA, Taylor DW (1994) Formation of two-dimensional complexes of F-actin and crosslinking proteins on lipid monolayers: demonstration of unipolar alpha-actinin-F-actin crosslinking. Biophys J 67: 1976-1983. doi:10.1016/S0006-3495(94)80680-0. PubMed: 7858134. 7858134 10.1016/S0006-3495(94)80680-0 PMC1225572

[41] Volkmann N, DeRosier D, Matsudaira P, Hanein D (2001) An atomic model of actin filaments cross-linked by fimbrin and its implications for bundle assembly and function. J Cell Biol 153: 947-956. doi:10.1083/jcb.153.5.947. PubMed: 11381081. 11381081 10.1083/jcb.153.5.947 PMC2174342

[42] Nagy A, Piszczek G, Sellers JR (2009) Extensibility of the extended tail domain of processive and nonprocessive myosin V molecules. Biophys J 97: 3123-3131. doi:10.1016/j.bpj.2009.09.033. PubMed: 20006949. 20006949 10.1016/j.bpj.2009.09.033 PMC2793355

[43] O’Shea EK, Klemm JD, Kim PS, Alber T (1991) X-ray structure of the GCN4 leucine zipper, a two-stranded, parallel coiled coil. Science 254: 539-544. doi:10.1126/science.1948029. PubMed: 1948029. 1948029 10.1126/science.1948029

[44] Takagi Y, Yang Y, Fujiwara I, Jacobs D, Cheney RE et al. (2008) Human myosin Vc is a low duty ratio, nonprocessive molecular motor. J Biol Chem 283: 8527-8537. doi:10.1074/jbc.M709150200. PubMed: 18201966. 18201966 10.1074/jbc.M709150200

[45] Snyder GE, Sakamoto T, Hammer JA 3rd, Sellers JR, Selvin PR (2004) Nanometer localization of single green fluorescent proteins: evidence that myosin V walks hand-over-hand via telemark configuration. Biophys J 87: 1776-1783. doi:10.1529/biophysj.103.036897. PubMed: 15345556. 15345556 10.1529/biophysj.103.036897 PMC1304582

[46] Ishikawa K, Catlett NL, Novak JL, Tang F, Nau JJ et al. (2003) Identification of an organelle-specific myosin V receptor. J Cell Biol 160: 887-897. doi:10.1083/jcb.200210139. PubMed: 12642614. 12642614 10.1083/jcb.200210139 PMC2173761

[47] Sukow C, DeRosier D (1998) How to analyze electron micrographs of rafts of actin filaments crosslinked by actin-binding proteins. J Mol Biol 284: 1039-1050. doi:10.1006/jmbi.1998.2211. PubMed: 9837725. 9837725 10.1006/jmbi.1998.2211

[48] Sukow C, DeRosier DJ (2003) Order, disorder, and perturbations in actin-aldolase rafts. Biophys J 85: 525-536. doi:10.1016/S0006-3495(03)74497-X. PubMed: 12829507. 12829507 10.1016/S0006-3495(03)74497-X PMC1303108

[49] De La Cruz EM, Wells AL, Sweeney HL, Ostap EM (2000) Actin and light chain isoform dependence of myosin V kinetics. Biochemistry 39: 14196-14202. doi:10.1021/bi001701b. PubMed: 11087368. 11087368 10.1021/bi001701b

[50] Arjonen A, Kaukonen R, Ivaska J (2011) Filopodia and adhesion in cancer cell motility. Cell Adhes Migration 5: 421-430. doi:10.4161/cam.5.5.17723. PubMed: 21975551. 10.4161/cam.5.5.17723 PMC3218609 21975551

[51] Khurana S, George SP (2011) The role of actin bundling proteins in the assembly of filopodia in epithelial cells. Cell Adhes Migration 5: 409-420. doi:10.4161/cam.5.5.17644. PubMed: 21975550. 10.4161/cam.5.5.17644 PMC3218608 21975550

[52] Ali MY, Previs SB, Trybus KM, Sweeney HL, Warshaw DM (2013) Myosin VI has a one track mind versus myosin Va when moving on actin bundles or at an intersection. Traffic 14: 70-81. PubMed: 23046080. 23046080 10.1111/tra.12017 PMC3548028

[53] Oke OA, Burgess SA, Forgacs E, Knight PJ, Sakamoto T et al. (2010) Influence of lever structure on myosin 5a walking. Proc Natl Acad Sci U S A 107: 2509-2514. doi:10.1073/pnas.0906907107. PubMed: 20133809. 20133809 10.1073/pnas.0906907107 PMC2823865

