# Supplementary figures and images for: Actin Structure-Dependent Stepping of Myosin 5a and 10 during Processive Movement
Source: PLoS One. 2013 Sep 19;8(9):e74936. doi: 10.1371/journal.pone.0074936 (PMC3777900; doi:10.1371/journal.pone.0074936)

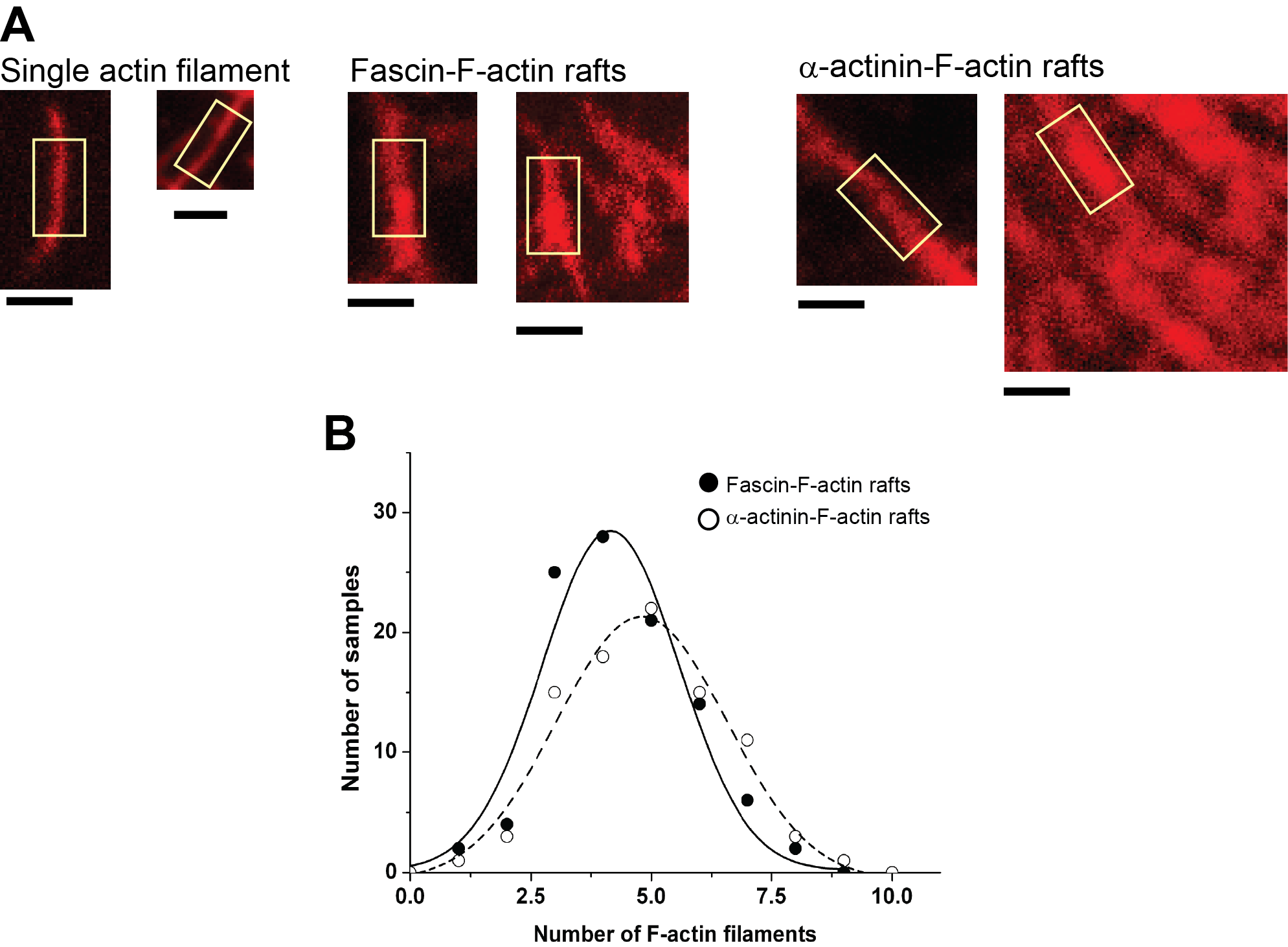

Supplement: Figure S1 — Fluorescence intensity measurement of fascin- and α-actinin-F-actin rafts. (A) Actin filaments were labeled with rhodamine-phalloidin. The total fluorescence in the rectangle region of interest (white rectangle) was measured and from the background fluorescence was subtracted to get the fluorescence intensity of a single actin filament. The intensities of fascin- and α-actinin-F-actin rafts were normalized to that of a single actin filament. (B) Histogram of the normalized intensity of fascin- (closed circle) and α-actinin- (open circle)-F-actin rafts. The solid (fascin-F-actin rafts) and dashed (α-actinin-F-actin rafts) lines represent the fit with single Gaussian curve. The average number of actin filaments in fascin- and α-actinin-F-actin rafts were 3.98 ± 1.8 (n = 102, mean ± S.D.) and 4.88 ± 1.85 (n = 89, mean ± S.D.), respectively. (TIF) [file pone.0074936.s001.tif]

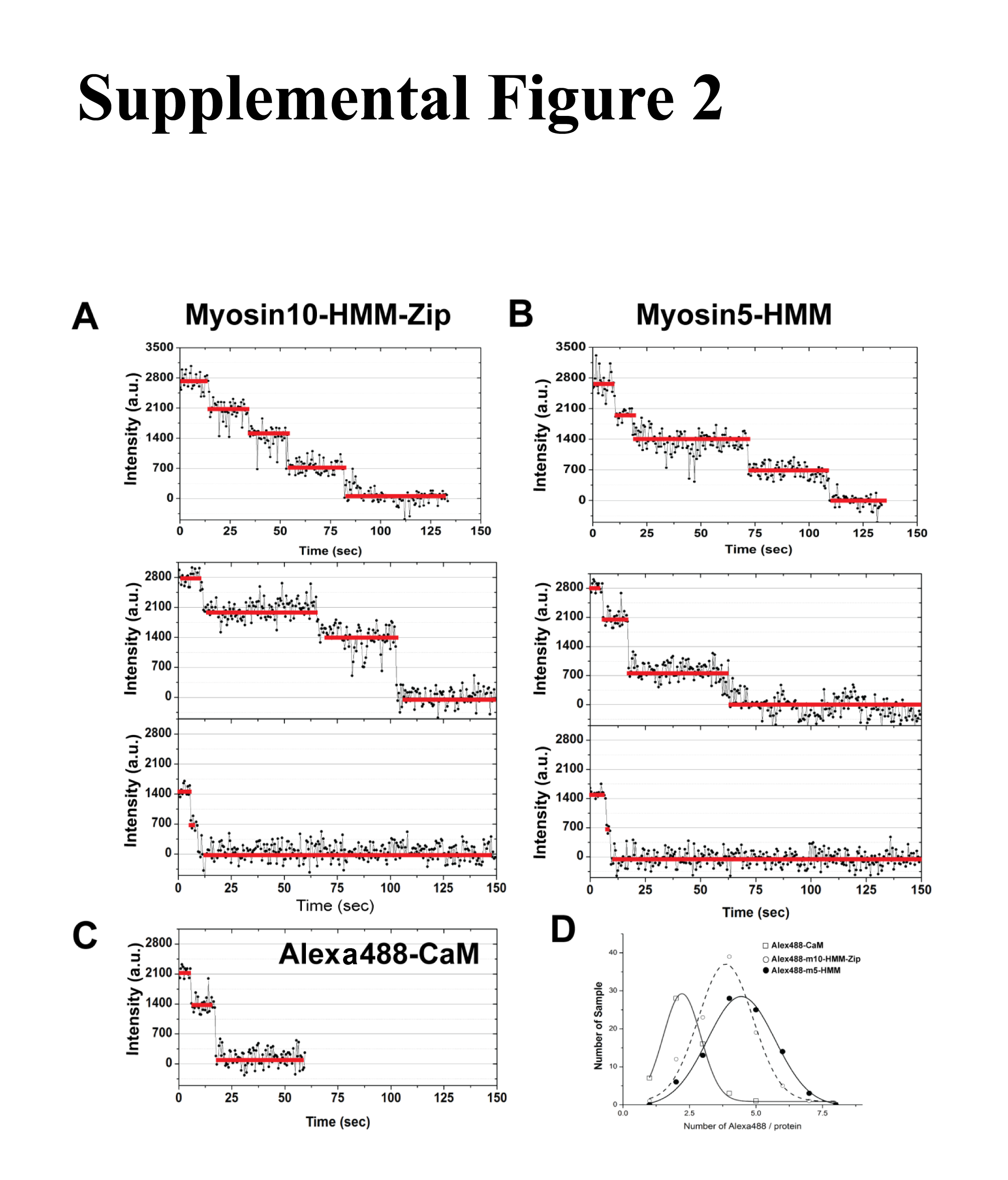

Supplement: Figure S2 — Determination of the number of Alexa Fluor 488 dyes per HMM molecule. Individual molecules of Alexa488-CaM-m10-HMM-Zip (A), Alexa488-CaM-m5-HMM (B), and Alexa488-labeled calmodulin (C) were bound on a cover glass surface and imaged by TIRF microscope. The photo-bleaching events were observed as a function of time. Step-wise photo-bleaching events were observed (red lines). (D) Histogram of the number of photo-bleaching events per molecule of m10-HMM-Zip, m5-HMM, and CaM. Data were fit with a single Gaussian curve. The average number of Alexa Fluor 488 on m10-HMM-Zip, m5-HMM, and CaM are 3.9 ± 2.4 and 4.4 ± 2.9, and 1.9, respectively. (TIF) [file pone.0074936.s002.tif]

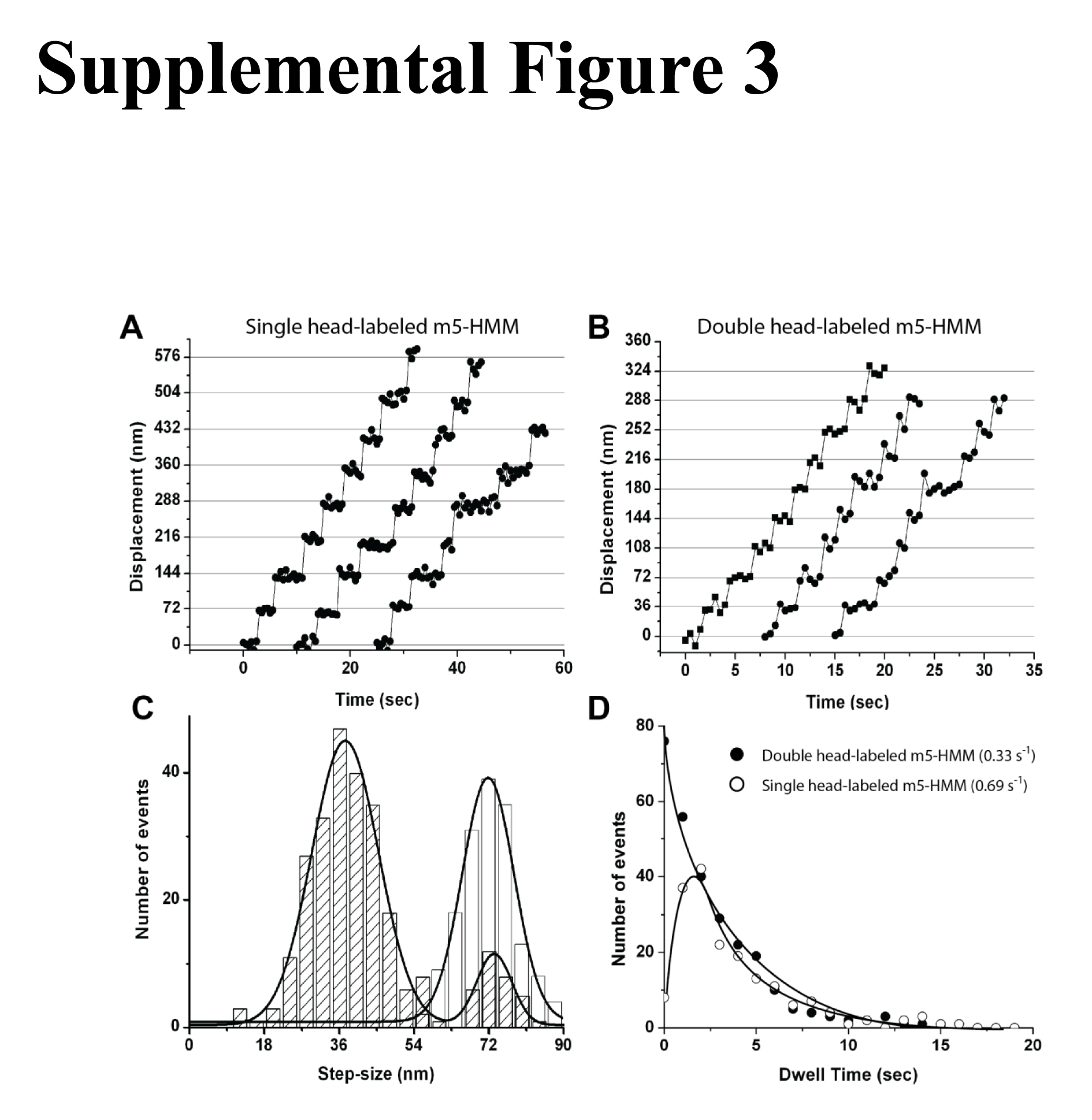

Supplement: Figure S3 — Single molecule analysis of m5-HMM stepping along single actin filaments. (A and B) Stepping traces of single- and double-head labeled m5-HMM, respectively. (C) Step size histogram of m5-HMM. The data of single-head-labeled m5-HMM (open bar) were fit with single Gaussian curve. The average step size is 72 ± 9 nm (n = 175, mean ± S.D.) The data of double-head labeled m5-HMM (right-hand shade) were fit with the sum of two Gaussians. The major and minor peaks are 36.5 ± 12 nm and 72.8 ± 4.7 nm (n = 270, mean ± S.D.), respectively. (D) The dwell time distribution of single- (open circle) and double- (closed circle) head labeled m5-HMM. The dwell time of single-head-labeled m5-HMM (0.21 s-1) was half of double-head labeled m5-HMM (0.43 s-1). The method for the fitting curves was described in Figure 2 D. (TIF) [file pone.0074936.s003.tif]

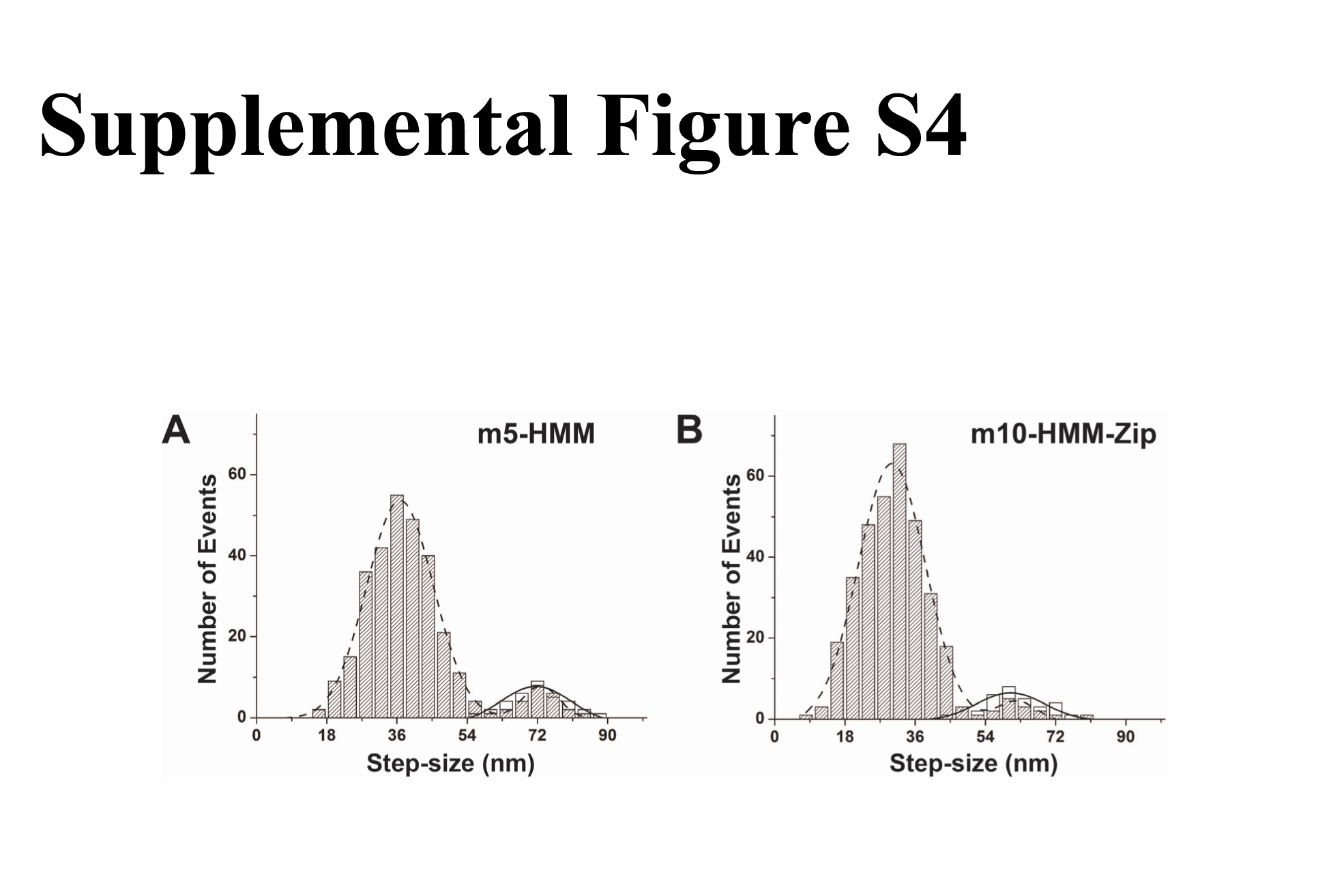

Supplement: Figure S4 — Step-size histograms of m5-HMM and m10-HMM-Zip with high exchang ratio of Alexa488-CaM to myosin. The step-size of myosin 5 and myosin 10 at low ATP concentration was measured under high exchange ratio of Alexa488-CaM to myosin molecule (30:1). (A) The step-size histogram of the double- (n = 308, right-hand shade) and single-head-labeled (n = 28, open bar) m5-HMM. The data of single- and double-head-labeled m5-HMM were fit with single Gaussian (solid line) and the sum of two Gaussians (dashed line), respectively. The average step-size of single-head-labeled m5-HMM is 72 ± 6.7 nm. The major and minor peaks of step-sizes of double-head-labeled m5-HMM step-size are 36.8 ± 5.2 nm and 73.3 ± 7.0 (mean ± S.D.). (B) The histogram of the step-size of double- (n = 344, right-hand shade) and single- head-labeled m10-HMM-Zip (n = 33, open bar). The data of single- and double-head-labeled m10-HMM-Zip were fit with single Gaussian curve (solid line) and the sum of two Gaussians (dashed line), respectively. The average step-size for double-head-labeled m10-HMM-Zip is 60.8 ± 3.3 nm. The major and minor step-size of single-headed-labeled m10-HMM-Zip are 30.2 ± 4.2 nm and 61.0 ± 5.6 nm, respectively. (TIF) [file pone.0074936.s004.tif]

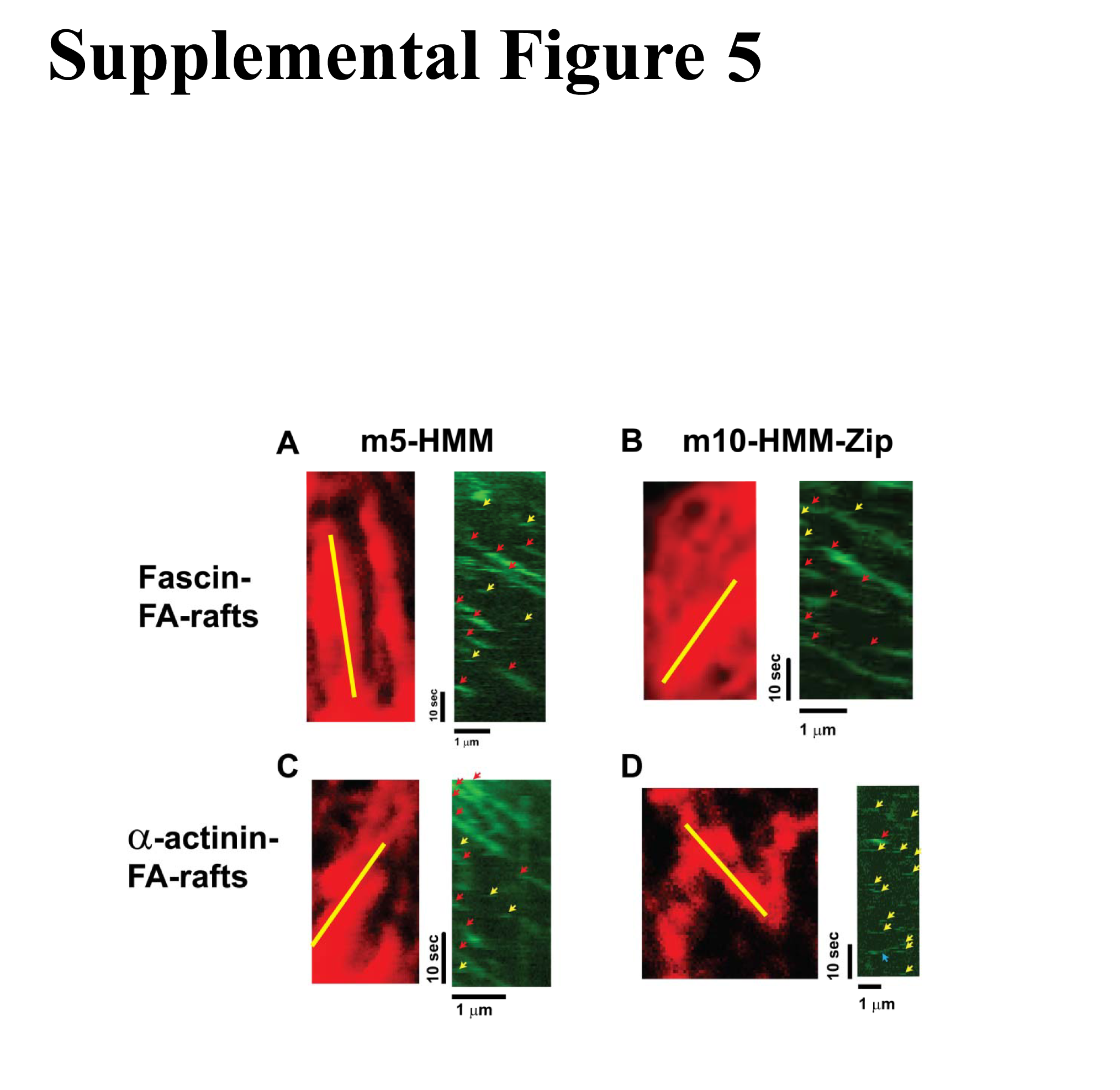

Supplement: Figure S5 — Movement of m5-HMM and m10 HMM-Zip on fascin-F-actin and α-actinin-F-actin rafts. In all panels the left image shows 2D bundle of F-actin labeled with rhodamine phalloidin and colored red. The yellow lines in each of these images were analyzed for the kymographs which are shown in the right panels. (A and B) Fluorescence images of fascin-F-actin rafts and kymographs showing movement of m5-HMM (A) and m10-HMM-Zip (B) (C and D) Fluorescence images of α-actinin-F-actin rafts and kymographs showing movements of m5-HMM (C) and m10-HMM-Zip (D). In the kymograph, red arrowheads represent processive movement and yellow arrowheads represent non-processive movement, i.e. where the myosin molecule bound and dissociated without significant movement. (TIF) [file pone.0074936.s005.tif]
